# Preconception hypoglycemia and adverse pregnancy outcomes in Chinese women aged 20–49 years: A retrospective cohort study in China

**DOI:** 10.1371/journal.pmed.1004667

**Published:** 2025-07-29

**Authors:** Hanbin Wu, Ying Yang, Chuanyu Zhao, Xinyi Lyu, Jiaxin Li, Jueming Lei, Meiya Liu, Xuan Hu, Yuzhi Deng, Yuan He, Yuanyuan Wang, Zuoqi Peng, Ya Zhang, Hongguang Zhang, Qiaomei Wang, Haiping Shen, Yiping Zhang, Donghai Yan, Ronald Ching Wan Ma, Chi Chiu Wang, Xu Ma

**Affiliations:** 1 Department of Obstetrics and Gynaecology, Prince of Wales Hospital, The Chinese University of Hong Kong, Hong Kong SAR, China; 2 National Research Institute for Family Planning, Beijing, China; 3 National Human Genetic Resource Center, Beijing, China; 4 Graduate School of Peking Union Medical College, Beijing, China; 5 Department of Maternal and Child Health, National Health Commission of the PRC, Beijing, China; 6 Li Ka Shing Institute of Health Science, Li Ka Shing Medical Sciences Building, Prince of Wales Hospital, The Chinese University of Hong Kong, Hong Kong SAR, China; 7 Department of Medicine and Therapeutics, Prince of Wales Hospital, The Chinese University of Hong Kong, Hong Kong SAR, China; 8 Hong Kong Institute of Diabetes and Obesity, Prince of Wales Hospital, The Chinese University of Hong Kong, Hong Kong SAR, China; 9 School of Biomedical Sciences, The Chinese University of Hong Kong, Hong Kong SAR, China; University of Leeds, UNITED KINGDOM OF GREAT BRITAIN AND NORTHERN IRELAND

## Abstract

**Background:**

In addition to hyperglycemia, women with hypoglycemia identified during pregnancy have a higher risk of adverse pregnancy outcomes. However, there is limited evidence of the association between prepregnant hypoglycemia and adverse pregnancy outcomes in women without pre-existing diabetes. This study aims to explore the association between maternal preconception hypoglycemia and adverse pregnancy outcomes among childbearing-aged women in China.

**Methods and findings:**

This was a retrospective cohort study of the National Free Preconception Checkup Project (NFPCP), including women who were aged 20–49, successfully conceived within one year without multiple gestations, and had complete information on pregnancy outcomes. Maternal fasting plasma glucose (FPG) concentrations were analyzed in the preconception examination stage, and women were divided into normal (FPG 3.9 to <5.6 mmol/L) and hypoglycemia (FPG < 3.9 mmol/L) groups. Adverse pregnancy outcomes included medical abortion, miscarriage or early stillbirth, preterm birth (PTB), macrosomia, low birth weight (LBW), large for gestational age (LGA), small for gestational age (SGA), birth defects, and perinatal death. Baseline characteristics of the two groups were balanced using inverse probability treatment weighting (IPTW) based on propensity scores. Both multivariable-adjusted and IPTW odds ratios (ORs) and 95% confidence intervals (CIs) were calculated to assess the association between preconception hypoglycemia and adverse pregnancy outcomes. Models adjusted for maternal age, ethnicity, educational level, occupation, region of gross domestic product, smoking, passive smoking, alcohol consumption, maternal preconception body mass index (BMI), parity, history of adverse pregnancy outcome, preconception medicine use, folic acid intake, diabetes, hypertension, anemia, thyroid disorder, liver disorder, and infection. ORs of adverse pregnancy outcomes with preconception hypoglycemia stratified by BMI were also reported. Among 4,866,919 women who participated in NFPCP during 2013–2016, 239,128 (4.91%) had preconception hypoglycemia. Compared to the normal group, women with preconception hypoglycemia had increased IPTW-multivariate adjusted ORs of PTB by 10% (95% CI [1.08, 1.12], *P* < 0.001), LBW by 8% (95% CI [1.03, 1.12], *P* = 0.001), SGA by 7% (95% CI [1.05, 1.08], *P* < 0.001), and birth defects by 21% (95% CI [1.06, 1.37], *P* = 0.004), while the ORs of medical abortion decreased by 6% (95% CI [0.91, 0.98], *P* = 0.002), miscarriage or early stillbirth by 5% (95% CI [0.92, 0.97], *P* < 0.001), macrosomia by 12% (95% CI [0.86, 0.90], *P* < 0.001), and LGA by 12% (95% CI [0.86, 0.89], *P* < 0.001) if mothers had a preconception hypoglycemia. The associations of maternal preconception hypoglycemia and adverse pregnancy outcomes varied among BMI groups. Among underweight women, preconception hypoglycemia was associated with a lower risk of medical abortion, miscarriage or early stillbirth, LGA, and PID, while overweight women had a lower risk of macrosomia and LGA. Moreover, a higher risk of miscarriage or early stillbirth and PTB was observed in obesity and underweight, respectively, in association with preconception hypoglycemia. Main limitations in the current study included the limited generalizability in other countries with varying disparities in healthcare and the lack of information on certain potential confounders (such as gestational complications and whether they received any related intervention after preconception examination).

**Conclusion:**

Preconception hypoglycemia was significantly associated with adverse pregnancy outcomes, and maternal preconception BMI could modify the association. In addition to paying attention to women with preconception hyperglycemia, our findings call for increased concern for women with hypoglycemia in preconception glycemic screening, with consideration of modified effects by preconception BMI, which might be worth exploring as a means to reduce adverse pregnancy outcomes.

## Author summary

**Why was this study done?:**

- Prior studies found that hypoglycemia identified during pregnancy was associated with adverse maternal and neonatal outcomes, but the association of maternal preconception hypoglycemia with adverse pregnancy outcomes remains unclear.
- A previous study found that childbearing-aged women with elevated fasting plasma glucose prior to pregnancy have increased risks of adverse pregnancy outcomes, but they didn’t separate women with fasting plasma glucose (FPG) levels <3.9 mmol/L from the normal group and evaluate the effect of preconception hypoglycemia on adverse pregnancy outcomes.
- Little is known about whether maternal preconception hypoglycemia could increase the risks of various adverse pregnancy outcomes in Chinese childbearing-aged women.

**What did the researchers do and find?:**

- We assessed whether preconception hypoglycemia was associated with adverse pregnancy outcomes in childbearing-aged women (*N* = 4,866,919), and further evaluated the modifying effect of maternal preconception body mass index (BMI) on the associations.
- Compared to women with normal FPG levels, women with preconception hypoglycemia were relatively young and had a higher proportion of underweight status and anemia.
- Women with preconception hypoglycemia had higher risks of adverse pregnancy outcomes, including preterm birth, low birth weight, small for gestational age, and birth defects, than those with normal FPG levels, and maternal preconception BMI modified the associations between preconception hypoglycemia and adverse pregnancy outcomes.

**What do these findings mean?:**

- In addition to elevated FPG prior to pregnancy, further attention should be paid to women with preconception hypoglycemia, which could also increase the risk of adverse pregnancy outcomes.
- Comprehensive screening and management for preconception FPG status, whether hyperglycemia or hypoglycemia, in childbearing-aged women, with consideration of modified effects by preconception BMI, is essential for early intervention to improve pregnancy outcomes. Further studies might be needed to determine whether improving nutritional status for women with preconception hypoglycemia reduces the risk of subsequent adverse pregnancy outcomes.
- The limitations of this study are limited generalizability in other countries with varying disparities in healthcare and the lack of information on certain potential confounders (such as gestational complications and whether they received any related intervention after preconception examination).

## Abstract 3

In a retrospective cohort study, Hanbin Wu and colleagues investigated whether preconception hypoglycemia increases the risk of adverse pregnancy outcomes in a Chinese population.

## Introduction

Glucose, one of the essential body nutrients for maintaining homeostasis, is closely regulated to maintain a physiological range for normal body functions [1–3]. Elevated blood glucose levels, or hyperglycemia (including prediabetes and type 2 diabetes), increase the risk of developing cardiovascular disease, chronic kidney disorders, and other metabolic comorbidities, such as dyslipidemia and obesity [4]. On the other hand, two meta-analysis studies and five observational studies involving diverse ethnicities and sample sizes found that low blood glucose levels, or hypoglycemia, in individuals without pre-existing diabetes or cardiovascular diseases, are associated with an increased risk of new-onset diabetes and all-cause mortality [5–11].

Maintaining an optimal glucose level in women during preconception and gestational periods is crucial, as it could reduce the risk of adverse pregnancy outcomes. Substantial evidence, including a multi-center prospective observational study with over 25 thousand participants and a large population-based retrospective study with over 6 million in China, demonstrates that hyperglycemia, identified prior to or during pregnancy, is associated with increased risks of adverse pregnancy outcomes [12,13], and early screening and management of hyperglycemia are essential to reduce adverse pregnancy outcomes [4,14–18]. In addition to hyperglycemia, some studies reported that maternal hypoglycemia identified during pregnancy might lead to decreased levels of human placental lactogen and reduced fetal insulin levels, creating an unfavorable environment for fetal growth and increasing the risk of adverse pregnancy outcomes, such as pre-eclampsia, fetal growth retardation, low birth weight (LBW), and low Apgar score [19–29]. With the extensive evidence, the implications of gestational hypoglycemia have been recognized, but only two studies thus far have explored the association of maternal preconception hypoglycemia with adverse pregnancy outcomes. Zeng and colleagues found that maternal preconception hypoglycemia (fasting plasma glucose [FPG] < 3.9 mmol/L) was only associated with spontaneous abortion in over 60 thousand participants, while Xu and colleagues found a significant association of maternal preconception hypoglycemia (FPG < 2.8 mmol/L) with preterm birth (PTB) in around 5 million participants [30,31]. With the limited evidence and conflicting results, the associations between maternal preconception hypoglycemia and adverse pregnancy outcomes remain unclear. A comprehensive study with a large sample size and adherence to the American Diabetes Association (ADA) criteria for hypoglycemia might be essential to provide conclusive evidence.

Hence, we conducted a retrospective population-based cohort study based on the National Free Preconception Checkups Project (NFPCP) in China, involving around 4.8 million childbearing-aged women, to evaluate the association between preconception hypoglycemia and adverse pregnancy outcomes.

## Methods

### Data source

The data used in this retrospective cohort study were obtained from the NFPCP, a national free health service for reproductive-aged women who plan to conceive in mainland China, which provides preconception examinations and counseling. NFPCP was initiated by the National Health Commission and the Ministry of Finance of the People’s Republic of China in 2010 and began serving only rural married spouses within 220 counties in 31 provinces from 2010 to 2012, and was further expanded to urban married spouses with 2,907 counties in mainland China after 2013. NFPCP includes preconception examination, early pregnancy follow-up, and pregnancy outcome follow-up. During the preconception examination, couples (women aged 20–49 years old; no age limitation was set for men) willing to conceive within the next 6 months were encouraged to participate in the project. Baseline information, including demographic characteristics, lifestyle, history of chronic diseases, and reproductive history, was collected through a face-to-face interview by trained health staff in the local maternal and child healthcare service centers using a standard and structured questionnaire. Body weight and height of participants wearing light, indoor clothes, and no shoes were measured. Seated blood pressure was measured in the right arm using an automated blood pressure monitor on a single occasion after participants rested for ≥10 min. For those couples who are willing to participate in the preconception examination, blood glucose screening is universal and recommended by the guideline of hyperglycemia in pregnancy [32]. Blood samples after an overnight fast for at least 8 hours were taken and immediately stored at 4–8 °C and then sent to the local laboratories. All data were uploaded and transferred remotely and stored in the NFPCP medical service information system, supported by the National Research Institute for Health and Family Planning.

**Fig 1:**
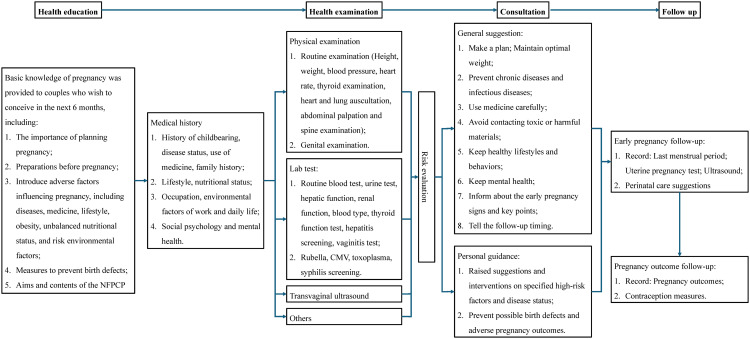
Detailed design, organization, and implementation of the NFPCP. Abbreviation: NFPCP, National Free Preconception Checkup Project; CMV, cytomegalovirus.

After the preconception examination, all participants were followed up by trained local health staff via telephone. The first interview was conducted within 3 months after the examination to track their pregnancy status and record the last menstrual period. If participants did not get pregnant, repeated inquiries were conducted within the next 3 months until 1 year after the baseline examination. Participants who didn’t conceive successfully within 1 year after the preconception examination were considered infertile and not followed further. Participants who had become pregnant were interviewed again for pregnancy outcomes within 1 year after completing the first follow-up, and information about the current pregnancy outcomes, delivery dates, and neonatal conditions was recorded. Details about the NFPCP-related design, organization, and implementation can be found elsewhere [33]. The flowchart of the detailed design, organization, and implementation of the NFPCP is shown in Fig 1. This project was approved by the Institutional Review Board at the National Research Institute for Family Planning (**IRB-201001**) in Beijing, China, and conducted according to the guidelines of the Declaration of Helsinki. Written informed consent was obtained from all participants at the beginning of the preconception examinations. The analysis was not planned before the study. It started in August 2023, and additional analyses were conducted from February to March 2025 in response to suggestions from journal reviews.

**Fig 2:**
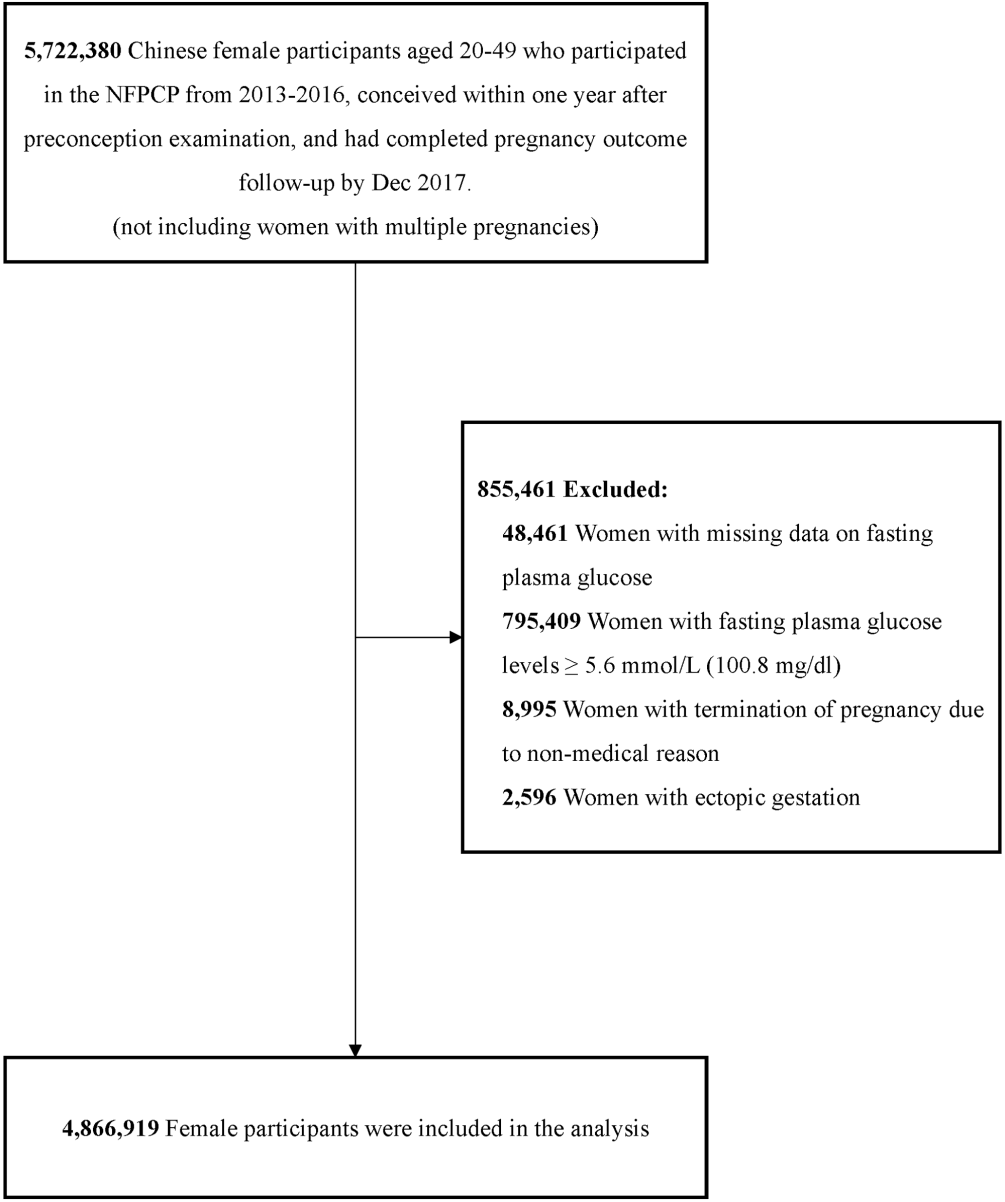
Flowchart of study population selection. Abbreviation: NFPCP, National Free Preconception Checkup Project.

This study followed the Strengthening the Reporting of Observational Studies in Epidemiology (**STROBE**) reporting guideline (S1 STROBE checklist). Initially, we included 5,722,380 Chinese women aged 20–49 years who participated in NFPCP from January 2013 to December 2016, successfully conceived within one year without multiple gestations, and had completed information on pregnancy outcomes by December 2017. Those women who had missing data on preconception FPG results (*n* = 48,461) and FPG level ≥ 5.6 mmol/L (≥100.8 mg/dl) (*n* = 795,409) were excluded. Those women who terminated pregnancy due to non-medical reasons (e.g., divorce) (*n* = 8,995) and had ectopic pregnancy (due to missing associated risk factors) (*n* = 2,596) were also excluded. Finally, 4,866,919 women with preconception FPG < 5.6 mmol/L (<100.8 mg/dl) were included in the analysis. The flowchart of the study population selection is shown in Fig 2.

### Biochemical test

Maternal preconception FPG concentrations were measured utilizing glucose oxidase or hexokinase methods in the local laboratories [13]. The National Center of Clinical Laboratories for Quality Inspection and Detection was responsible for the external quality assessment and was monitored biannually. To evaluate the association of maternal preconception FPG with adverse pregnancy outcomes, the participants were divided into two groups based on the ADA standards [34]: normal group with FPG 3.9 to <5.6 mmol/L (70.2 to <100.8 mg/dl) and hypoglycemia group with FPG < 3.9 mmol/L (<70.2 mg/dl).

### Outcomes

The adverse pregnancy outcomes included (1) medical abortion, defined as the termination of surgical methods due to eugenics or illness pregnancy; (2) miscarriage or early gestational stillbirth, including spontaneous abortion and unintentional fetal death before 28 weeks of gestation [35]; (3) PTB, defined as delivery at gestational ages between 28 and <37 weeks; (4) macrosomia, defined as newborn birth weight ≥ 4,000g; (5) LBW, defined as newborn birth weight < 2,500g; (6) large for gestational age (LGA), defined as a newborn birth weight above the 90th percentile for the gestational age and baby’s sex; (7) small for gestational age (SGA), defined as a newborn birth weight below the 10th percentile for the gestational age and baby’s sex; (8) birth defects, defined as major abnormalities in the fetus occurring before birth, such as Trisomy 21, left cleft lip, cleft palate, anencephaly, cerebrospinal meningitis, hydrocephalus, open spina bifida, and congenital heart disease; and (9) perinatal death, defined as stillbirth after 28 weeks of gestation or neonatal death after birth within 7 days.

### Covariates

Maternal demographic characteristics and preconception clinical factors were included in our study as important potential confounders. Demographic covariates included maternal age, ethnicity (Han or others), education (illiterate, primary school, junior high school, senior high school, junior college and (or) undergraduate, postgraduate), occupation (farmer or others), active smoking (yes or no), passive smoking (yes or no), alcohol consumption (yes or no), and region of gross domestic product (GDP) per capita (≤40,000; 40,001–50,000; 50,001–70,000; >70,000 Chinese yuan (CNY)/year). Clinical covariates included maternal body mass index (BMI) (underweight [<18.5 kg/m^2^], normal [18.5–23.9 kg/m^2^], overweight [24.0–27.9 kg/m^2^], and obese [≥28.0 kg/m^2^] by Chinese criteria) [36,37], parity (nulliparous or multiparous), history of adverse pregnancy outcomes including stillbirth, induced abortion, birth defect, or PTB in previous pregnancies (yes or no), preconception medicine use (yes or no), folic acid intake (yes or no), diabetes (yes or no), hypertension (yes or no), anemia (yes or no), thyroid disorder (yes or no), liver disorder (yes or no), and infection status (including Neisseria gonorrhoeae, Chlamydia trachomatis, Toxoplasma gondii, Cytomegalovirus, Treponema pallidum, Rubella virus, or Hepatitis B virus). Details about the covariates’ definition are shown in the S1 Table. The definition of covariates has been reported previously [13,38].

As included covariates with missing values of no more than 5% could be assumed to be missing at random, a new label, missing, was generated for those variables with missing values.

### Statistical analysis

The directed acyclic graph (DAG), a type of causal diagram, was used to identify variables that could confound the association between maternal preconception hypoglycemia and risk of adverse pregnancy outcomes [39,40]. A minimal sufficient adjustment set (MSAS) was selected as a priori potential confounders using the DAGitty online (S1 Fig) [41]. The MSAS included maternal age, ethnicity, educational level, occupation, region of GDP, smoking, passive smoking, alcohol consumption, maternal preconception BMI, parity, history of adverse pregnancy outcome, preconception medicine use, folic acid intake, diabetes, hypertension, anemia, thyroid disorder, liver disorder, and infection. The inverse probability treatment weighting (IPTW) method based on propensity scores was used to balance the difference in baseline characteristics between two groups (Hypoglycemia and Normal) [42]. The between-group equilibrium of all covariates was assessed using the standardized mean difference (SMD) (Table 1).

**Table 1:** Baseline characteristics of the study population.

Maternal characteristics	Overall	Hypoglycemia	Normal group	SMD	
		<3.9 mmol/L	3.9 to <5.6 mmol/L	Before weighted	After weighted
	(*n* = 4,866,919)	(*n* = 239,128)	(*n* = 4,627,791)		
**Age, year [median (IQR)]**	26 (24, 29)	25 (23, 28)	26 (24, 29)	0.1651	0.1467
**Ethnicity, *n*(%)**				0.1927	0.1721
Han	4,461,041 (91.66%)	206,229 (86.24%)	4,254,812 (91.94%)		
Others	340,951 (7.01%)	29,526 (12.35%)	311,425 (6.73%)		
Missing value	64,927 (1.33%)	3,373 (1.41%)	61,554 (1.33%)		
**Education level, *n*(%)**				0.0538	0.0485
Illiterate	11,672 (0.24%)	609 (0.25%)	11,063 (0.24%)		
Primary school	138,630 (2.85%)	8,802 (3.68%)	129,828 (2.81%)		
Junior high school	2,774,509 (57.01%)	133,710 (55.92%)	2,640,799 (57.06%)		
Senior high school	903,651 (18.57%)	45,064 (18.85%)	858,587 (18.55%)		
Junior college and (or) undergraduate	866,065 (17.79%)	42,824 (17.91%)	823,241 (17.79%)		
Postgraduate	25,898 (0.53%)	1,412 (0.59%)	24,486 (0.53%)		
Missing value	146,494 (3.01%)	6,707 (2.80%)	139,787 (3.02%)		
**Occupation, *n*(%)**				0.0616	0.0529
Farmer	3,432,564 (70.53%)	162,230 (67.84%)	3,270,334 (70.67%)		
Others	1,275,632 (26.21%)	68,661 (28.71%)	1,206,971 (26.08%)		
Missing value	158,723 (3.26%)	8,237 (3.44%)	150,486 (3.25%)		
**Region of GDP (CNY) per capita, *n*(%)**				0.1236	0.1096
GDP ≤ 40,000	2,403,691 (49.39%)	120,029 (50.19%)	2,283,662 (49.35%)		
40,000 < GDP ≤ 50,000	1,075,923 (22.11%)	61,761 (25.83%)	1,014,162 (21.91%)		
50,000 < GDP ≤ 70,000	1,101,180 (22.63%)	45,828 (19.16%)	1,055,352 (22.80%)		
GDP ≥ 70,000 CNY	286,125 (5.88%)	11,510 (4.81%)	274,615 (5.93%)		
**Smoking, *n*(%)**				0.0118	0.0104
No	4,832,358 (99.29%)	237,515 (99.33%)	4,594,843 (99.29%)		
Yes	10,631 (0.22%)	591 (0.25%)	10,040 (0.22%)		
Missing value	23,930 (0.49%)	1,022 (0.43%)	22,908 (0.50%)		
**Passive smoking, *n*(%)**				0.0056	0.0050
No	4,288,396 (88.11%)	210,565 (88.06%)	4,077,831 (88.12%)		
Yes	554,294 (11.39%)	27,449 (11.48%)	526,845 (11.38%)		
Missing value	24,229 (0.50%)	1,114 (0.47%)	23,115 (0.50%)		
**Alcohol consumption, *n*(%)**				0.0455	0.0397
No	4,700,034 (96.57%)	229,214 (95.85%)	4,470,820 (96.61%)		
Yes	138,570 (2.85%)	8,618 (3.60%)	129,952 (2.81%)		
Missing value	28,315 (0.58%)	1,296 (0.54%)	27,019 (0.58%)		
**Body mass index, *n*(%)**				0.1366	0.1202
Underweight, <18.5 kg/m^2^	669,635 (13.76%)	41,784 (17.47%)	627,851 (13.57%)		
Normal, 18.5 to <24.0 kg/m^2^	3,481,358 (71.53%)	169,723 (70.98%)	3,311,635 (71.56%)		
Overweight, 24.0 to <28.0 kg/m^2^	573,265 (11.78%)	22,569 (9.44%)	550,696 (11.90%)		
Obesity, ≥28.0 kg/m^2^	124,609 (2.56%)	4,303 (1.80%)	120,306 (2.60%)		
Missing value	18,052 (0.37%)	749 (0.31%)	17,303 (0.37%)		
**Parity, *n*(%)**				0.1313	0.1160
Nulliparous	2,964,120 (60.90%)	159,964 (66.89%)	2,804,156 (60.59%)		
Multiparous	1,902,799 (39.10%)	79,164 (33.11%)	1,823,635 (39.41%)		
**Previous history of adverse pregnancy outcomes, *n*(%)**				0.0116	0.0101
No	4,706,281 (96.70%)	231,701 (96.89%)	4,474,580 (96.69%)		
Yes	160,638 (3.30%)	7,427 (3.11%)	153,211 (3.31%)		
**Preconception medicine use**				0.0107	0.0090
No	4,682,695 (96.21%)	230,426 (96.36%)	4,452,269 (96.21%)		
Yes	159,331 (3.27%)	7,627 (3.19%)	151,704 (3.28%)		
Missing value	24,893 (0.51%)	1,075 (0.45%)	23,818 (0.51%)		
**Folic acid intake, *n*(%)**				0.0259	0.0224
No	992,530 (20.39%)	49,046 (20.51%)	943,484 (20.39%)		
Yes	3,818,704 (78.46%)	186,696 (78.07%)	3,632,008 (78.48%)		
Missing value	55,685 (1.14%)	3,386 (1.42%)	52,299 (1.13%)		
**Diabetes, *n*(%)**				0.0025	0.0022
No	4,866,538 (99.99%)	239,114 (99.99%)	4,627,424 (99.99%)		
Yes	381 (0.01%)	14 (0.01%)	367 (0.01%)		
**Hypertension, *n*(%)**				0.0170	0.0151
No	4,765,344 (97.91%)	234,356 (98.00%)	4,530,988 (97.91%)		
Yes	74,526 (1.53%)	3,277 (1.37%)	71,249 (1.54%)		
Missing value	27,049 (0.56%)	1,495 (0.63%)	25,554 (0.55%)		
**Anemia, *n*(%)**				0.1190	0.1040
No	4,488,527 (92.23%)	212,753 (88.97%)	4,275,774 (92.39%)		
Yes	367,668 (7.55%)	25,824 (10.80%)	341,844 (7.39%)		
Missing value	10,724 (0.22%)	551 (0.23%)	10,173 (0.22%)		
**Thyroid disorder, *n*(%)**				0.0635	0.0556
No	4,546,316 (93.41%)	219,744 (91.89%)	4,326,572 (93.49%)		
Yes	268,280 (5.51%)	16,621 (6.95%)	251,659 (5.44%)		
Missing value	52,323 (1.08%)	2,763 (1.16%)	49,560 (1.07%)		
**Liver disorder, n(%)**				0.0633	0.0553
No	4,623,207 (94.99%)	223,831 (93.60%)	4,399,376 (95.06%)		
Yes	239,813 (4.93%)	15,040 (6.29%)	224,773 (4.86%)		
Missing value	3,899 (0.08%)	257 (0.11%)	3,642 (0.08%)		
**Active infection, *n*(%)**				0.1121	0.0983
No	4,366,337 (89.71%)	206,572 (86.39%)	4,159,765 (89.89%)		
Yes	278,593 (5.72%)	16,835 (7.04%)	261,758 (5.66%)		
Missing value	221,989 (4.56%)	15,721 (6.57%)	206,268 (4.46%)		

Abbreviations: IQR, inter quantile range; GDP, gross domestic product; CNY, Chinese Yuan; SMD, standard mean difference.

Baseline characteristics were compared between the two groups for the unweighted and IPTW-weighted populations, respectively. Maternal age variables were expressed as medians and interquartile ranges (IQRs), and categorical variables were presented as numbers and percentages.

To explore the association between maternal preconception hypoglycemia and adverse pregnancy outcomes, the odds ratios (ORs) and 95% confidence intervals (CIs) were calculated by logistic regression using the normal group as reference, and two models were fitted: Model 1 was a crude model without adjusting any covariates, and Model 2 adjusted with MSAS. Both multivariable-adjusted ORs and IPTW-adjusted ORs were reported. To address the multiple comparison issues and maintain adequate statistical power, the Benjamini-Hochberg correction was used to control the false discovery rate (FDR) at 5% involved in evaluating the associations of maternal preconception hypoglycemia and nine adverse pregnancy outcomes.

Alongside treating maternal preconception FPG levels as a binary exposure by ADA standards, we further assess the dose–response relationship between maternal preconception FPG levels and adverse pregnancy outcomes using restricted cubic spline (RCS) analysis. Wald statistics were used to examine the non-linear trend, and the covariates adjusted in RCS regression were the same as those adjusted in Model 2.

ORs of adverse pregnancy outcomes with preconception hypoglycemia stratified by maternal preconception BMI were analyzed to determine modification effects, and the relative excess risk due to interaction (RERI) was further applied to determine the additive-scale interactions between maternal preconception hypoglycemia and BMI status. The 95% CIs of RERI were estimated by the delta method [43].

To enhance the stability of the results, sensitivity analysis was conducted by excluding participants with a history of adverse pregnancy outcomes or with a pre-existing diabetes. All the covariates adjusted in the model were mentioned in the S1 Table. Furthermore, we calculated the E-value, reflecting the sensitivity of the results to unmeasured confounding. The E-value is defined as the minimum unmeasured confounding effect required to completely subvert the OR in the study, controlling for the measured confounding factor [44,45].

All analyses were performed using R software, version 4.2.2 (R Foundation for Statistical Computing), with the analysis packages tidyverse, version 2.0.0; speedglm, version 0.3−5; rms, version 6.5−0; forestplot, version 3.1.1; patchwork, version 1.1.2; ggbreak, version 0.1.4; interactionR, version 0.1.6; Evalue, version 4.1.3; and ipw, version 1.2.1. All statistical tests were two-sided, and *P* < 0.05 was considered statistically significant.

## Results

### Baseline characteristics according to maternal preconception hypoglycemia status

In total, 239,128 (4.91%) women were confirmed with preconception hypoglycemia. The median age was 26 (IQR 24, 29) years, and the median length from participating in preconception examination to pregnancy was 2.21 (IQR 0.86, 4.73) months. The baseline characteristics of the included participants are shown in Table 1. The hypoglycemic group was characterized by a younger maternal age and a greater ethnic diversity. Furthermore, women in the hypoglycemic group demonstrated a higher proportion of nulliparity, underweight, and anemia when compared to women with normal preconception FPG.

### Risk of adverse pregnancy outcomes according to maternal preconception hypoglycemia status and fasting plasma glucose levels

The detailed incidence of each adverse pregnancy outcome for women in the normal FPG group and hypoglycemia group is shown in Table 2. Compared with normal FPG group, women with preconception hypoglycemia had significantly increased IPTW-multivariate adjusted ORs of PTB by 10% (95% CI [1.08, 1.12], *P* < 0.001), LBW by 8% (95% CI [1.03, 1.12], *P* = 0.001), SGA by 7% (95% CI [1.05, 1.08], *P* < 0.001), and birth defects by 21% (95% CI [1.06, 1.37], *P* = 0.004), but had decreased IPTW-multivariate adjusted ORs of medical abortion by 6% (95% CI [0.91, 0.98], *P* = 0.002), miscarriage or early stillbirth by 5% (95% CI [0.92, 0.97], *P* < 0.001), macrosomia by 12% (95% CI [0.86, 0.90], *P* < 0.001), and LGA by 12% (95% CI [0.86, 0.89], *P* < 0.001) were observed in women with preconception hypoglycemia, regardless the demographic and/or clinical covariates adjusted. However, no significant association between maternal preconception hypoglycemia and perinatal death was observed.

**Table 2:** Association between preconception hypoglycemia and adverse pregnancy outcomes.

Outcomes	Cases/participants (%)	*P*-value	Unweighted						IPTW					
			Unadjusted model		Full model		*E*-values		Unadjusted model		Full model		*E*-values	
			OR (95% CI)	*P*-value	OR (95% CI)	*P*-value	Point	CI	OR (95% CI)	*P*-value	OR (95% CI)	*P*-value	Point	CI
**Medical abortion**		<0.001												
3.9 to <5.6 mmol/L	64,929/4,627,791 (1.40%)		1.00 Reference	···	1.00 Reference	···			1.00 Reference	···	1.00 Reference	···		
<3.9 mmol/L	2,910/239,128 (1.22%)		0.87 (0.83, 0.90)	<0.001	0.94 (0.91, 0.98)	0.002	1.32	1.16	0.87 (0.84, 0.91)	<0.001	0.94 (0.91, 0.98)	0.002	1.32	1.16
**Miscarriage or early stillbirth**		<0.001												
3.9 to <5.6 mmol/L	129,365/4,627,791 (2.80%)		1.00 Reference	···	1.00 Reference	···			1.00 Reference	···	1.00 Reference	···		
<3.9 mmol/L	6,274/239,128 (2.62%)		0.94 (0.91, 0.96)	<0.001	0.94 (0.92, 0.97)	<0.001	1.32	1.21	0.94 (0.91, 0.96)	<0.001	0.95 (0.92, 0.97)	<0.001	1.29	1.21
**Preterm birth**		<0.001												
3.9 to <5.6 mmol/L	269,468/4,292,534 (6.28%)		1.00 Reference	···	1.00 Reference	···			1.00 Reference	···	1.00 Reference	···		
<3.9 mmol/L	15,427/221,500 (6.96%)		1.12 (1.10, 1.14)	<0.001	1.10 (1.08, 1.12)	<0.001	1.43	1.37	1.12 (1.10, 1.13)	<0.001	1.10 (1.08, 1.12)	<0.001	1.43	1.37
**Macrosomia**		<0.001												
3.9 to <5.6 mmol/L	217,473/4,280,371 (5.08%)		1.00 Reference	···	1.00 Reference	···			1.00 Reference	···	1.00 Reference	···		
<3.9 mmol/L	9,371/220,977 (4.24%)		0.83 (0.81, 0.85)	<0.001	0.88 (0.86, 0.90)	<0.001	1.53	1.46	0.83 (0.82, 0.85)	<0.001	0.88 (0.86, 0.90)	<0.001	1.53	1.46
**Low birth weight**		<0.001												
3.9 to <5.6 mmol/L	39,654/4,102,552 (0.97%)		1.00 Reference	···	1.00 Reference	···			1.00 Reference	···	1.00 Reference	···		
<3.9 mmol/L	2,260/213,866 (1.06%)		1.09 (1.05, 1.14)	<0.001	1.07 (1.03, 1.12)	0.001	1.34	1.21	1.09 (1.05, 1.14)	<0.001	1.08 (1.03, 1.12)	0.001	1.37	1.21
**Large for gestational age**		<0.001												
3.9 to<5.6 mmol/L	421,803/4,007,881 (10.52%)		1.00 Reference	···	1.00 Reference	···			1.00 Reference	···	1.00 Reference	···		
<3.9 mmol/L	18,044/204,822 (8.81%)		0.82 (0.81, 0.83)	<0.001	0.88 (0.86, 0.89)	<0.001	1.53	1.50	0.83 (0.81, 0.84)	<0.001	0.88 (0.86, 0.89)	<0.001	1.53	1.50
**Small for gestational age**		<0.001												
3.9 to <5.6 mmol/L	296,996/3,883,074 (7.65%)		1.00 Reference	···	1.00 Reference	···			1.00 Reference	···	1.00 Reference	···		
<3.9 mmol/L	17,584/204,362 (8.60%)		1.14 (1.12, 1.15)	<0.001	1.06 (1.05, 1.08)	<0.001	1.31	1.28	1.13 (1.11, 1.15)	<0.001	1.07 (1.05, 1.08)	<0.001	1.34	1.28
**Birth defects**		0.003												
3.9 to <5.6 mmol/L	4,013/4,627,791 (0.09%)		1.00 Reference	···	1.00 Reference	···			1.00 Reference	···	1.00 Reference	···		
<3.9 mmol/L	252/239,128 (0.11%)		1.22 (1.07, 1.38)	0.003	1.20 (1.06, 1.37)	0.005	1.69	1.31	1.22 (1.07, 1.38)	0.002	1.21 (1.06, 1.37)	0.004	1.71	1.31
**Perinatal death**		0.056												
3.9 to <5.6 mmol/L	15,389/4,627,791 (0.33%)		1.00 Reference	···	1.00 Reference	···			1.00 Reference	···	1.00 Reference	···		
<3.9 mmol/L	851/239,128 (0.36%)		1.07 (1.00, 1.15)	0.054	1.04 (0.97, 1.11)	0.316	1.24	1.00	1.07 (1.00, 1.14)	0.067	1.04 (0.97, 1.11)	0.315	1.24	1.00

Full model adjusted with maternal age, ethnicity, educational level, occupation, region of GDP, smoking, passive smoking, alcohol consumption, maternal preconception BMI, parity, history of adverse pregnancy outcome, preconception medicine use, folic acid use, diabetes, hypertension, anemia, thyroid disorder, liver disorder, and infection.

*P-value remains <0.05 after using the Benjamini–Hochberg correction with FDR = 0.05.

Abbreviations: IPTW, inverse probability treatment weighting; CI, confidence interval; OR, odds ratio; BMI, body mass index.

Given that similar results were observed in sensitivity analysis after excluding participants with a history of adverse pregnancy outcomes (S2 Table) and pre-existing diabetes (S3 Table), the effect of maternal preconception hypoglycemia on adverse pregnancy outcomes was not affected by these prior factors. To further assess the stability of associations, *E*-values were calculated, and an unmeasured confounder might need to be addressed with the association of preconception hypoglycemia and adverse pregnant outcomes for medical abortion (*E*-value = 1.32), miscarriage or early stillbirth (*E*-value = 1.29), PTB (*E*-value = 1.43), macrosomia (*E*-value = 1.53), LBW (*E*-value = 1.37), LGA (*E*-value = 1.53), SGA (*E*-value = 1.34), birth defects (*E*-value = 1.71), and perinatal death (*E*-value = 1.24), respectively.

### Dose–response association between maternal preconception fasting plasma glucose level and adverse pregnancy outcomes

**Fig 3:**
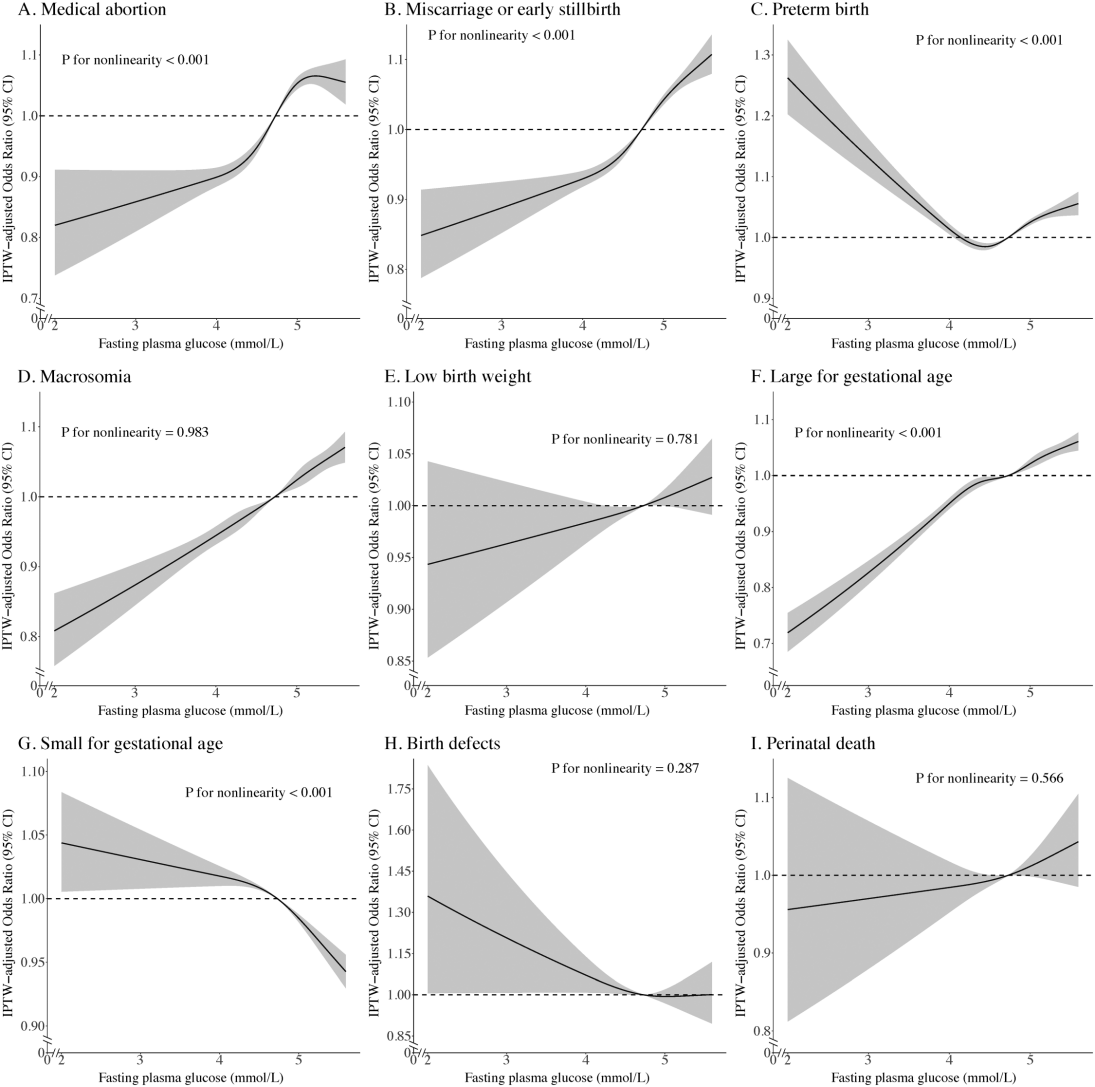
Dose-response relationship between maternal preconception fasting plasma glucose and risk of various adverse pregnancy outcomes (IPTW-multivariate adjusted models). The graph shows the IPTW-multivariate adjusted OR of association between maternal preconception FPG and the risk of adverse pregnancy outcomes. In the graph, black curves and shaded gray areas show predicted OR and 95% CI, respectively. Abbreviations: IPTW, inverse probability treatment weighting; OR, odds ratio; CI, confidence interval.

The RCS regressions revealed the dose-response association of maternal preconception FPG levels with various outcomes based on the IPTW-multivariate adjusted models (Fig 3). Nonlinear associations were found between preconception FPG and medical abortion (Fig 3A, *P* for nonlinear < 0.001), miscarriage or early stillbirth (Fig 3B, *P* for nonlinear = 0.012), PTB (Fig 3C, *P* for nonlinear < 0.001), LGA (Fig 3F, *P* for nonlinear < 0.001), and SGA (Fig 3G, *P* for nonlinear < 0.001). With the decrease in maternal preconception FPG, the risk of medical abortion, miscarriage or early stillbirth, and LGA gradually decreased, while the risk of PTB and SGA gradually increased. Conversely, the non-linear association was not observed between preconception FPG and macrosomia (Fig 3D, *P* for nonlinear = 0.983), LBW (Fig 3E, *P* for nonlinear = 0.781), birth defects (Fig 3H, *P* for nonlinear = 0.287), or perinatal death (Fig 3I, *P* for nonlinear = 0.566). With the decrease in maternal preconception FPG, the risk of birth defects gradually increased, but the risk of macrosomia, LBW, and perinatal death gradually decreased. Similar trends were also observed in the unweighted-multivariate adjusted models without IPTW (S2 Fig).

### Modification effect of maternal preconception BMI on the associations of maternal preconception hypoglycemia and adverse pregnancy outcomes

**Fig 4:**
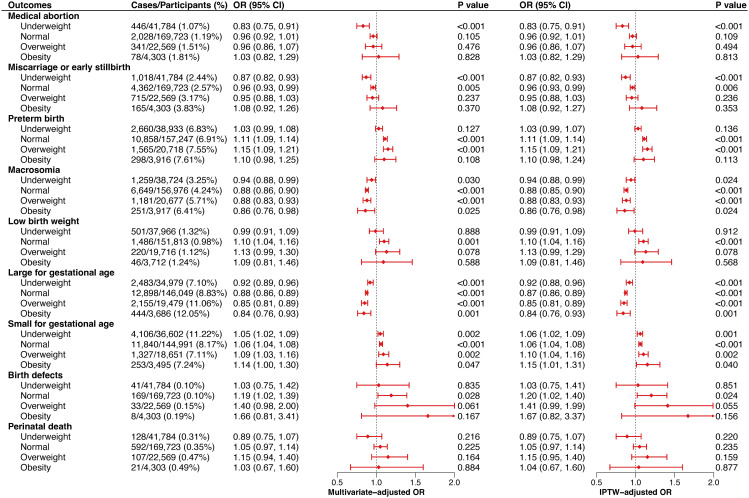
Associations between maternal preconception hypoglycemia and adverse pregnancy outcomes stratified by maternal preconception body mass index. Underweight, BMI < 18.5 kg/m^2^; Normal weight, BMI between 18.5 and 23.9 kg/m^2^; Overweight, BMI between 24.0 and 27.9 kg/m^2^; Obesity, BMI ≥ 28.0 kg/m^2^. Models were adjusted for maternal age, ethnicity, educational level, occupation, region, smoking, passive smoking, alcohol consumption, parity, history of adverse pregnancy outcome, preconception medicine use, folic acid use, diabetes, hypertension, anemia, thyroid disorder, liver disorder, and infection. Abbreviations: BMI, body mass index; IPTW, inverse probability treatment weighting; OR, odds ratio; CI, confidence interval.

As illustrated in Fig 4, the associations of maternal preconception hypoglycemia and adverse pregnancy outcomes varied by different preconception BMI statuses. Among women who were underweight prior to pregnancy, the effect of maternal preconception hypoglycemia on medical abortion and miscarriage or early stillbirth appeared to be increased, while its impact on macrosomia and LGA appeared to be relatively attenuated. Among women who were overweight prior to pregnancy, the effect of maternal preconception hypoglycemia on PTB appeared to be slightly increased. Moreover, the associations of maternal preconception hypoglycemia with SGA appeared to be relatively greater, but the association with LGA was diminished among women who were obese prior to pregnancy. Details are shown in the S4 Table. The interaction of preconception hypoglycemia and BMI status on adverse pregnancy outcomes can be found in S5 Table. Underweight women were observed to have a lower risk of medical abortion, miscarriage or early stillbirth, LGA, and PID associated with preconception hypoglycemia. A lower risk of macrosomia and LGA associated with preconception hypoglycemia was also observed in overweight women. Moreover, a higher risk of miscarriage or early stillbirth and PTB associated with preconception hypoglycemia was observed in obesity and underweight, respectively.

## Discussion

This population-based cohort study collected data from the NFPCP, involving over 4.7 million Chinese childbearing-aged women, to evaluate the association between maternal preconception hypoglycemia and adverse pregnancy outcomes. We found that 4.91% of participants had preconception FPG < 3.9 mmol/L, and women with preconception hypoglycemia might have increased risks of PTB, LBW, SGA, and birth defects, but have decreased risks of medical abortion, miscarriage or early stillbirth, macrosomia, and LGA. Additionally, we found nonlinear dose-response relationships between maternal preconception FPG and medical abortion, miscarriage or early stillbirth, PTB, LGA, and SGA. Furthermore, maternal preconception BMI status modified the associations between preconception hypoglycemia and adverse pregnancy outcomes.

Some observational studies have shown that hypoglycemia, identified during pregnancy after a glucose tolerance test in late pregnancy, increased the risk of gestational complications and adverse pregnancy outcomes, with the prevalence ranging from 3.7% to 31.8% [21–29]. To our knowledge, only two studies have yet reported the elusive effects of maternal preconception hypoglycemia on adverse pregnancy outcomes in the Chinese population. Zeng and colleagues only observed a significant association between maternal preconception hypoglycemia (defined as FPG < 3.9 mmol/L) and spontaneous abortion, which was not observed in PTB, among over 60 thousand participants, of which 5.06% had preconception hypoglycemia [30]. In a study with around 5 million participants, of whom 0.22% had hypoglycemia prior to pregnancy, Xu and colleagues observed a significant association between maternal preconception hypoglycemia (FPG < 2.8 mmol/L) and PTB [31]. The conflicting findings regarding the association between maternal preconception hypoglycemia and PTB might be attributed to the variations in population selection and different definitions of hypoglycemia and reference groups [30,31]. In our study, women with preconception hypoglycemia (4.91%) had an increased risk of PTB, aligning with Xu and colleagues’ findings [31]. Besides PTB, women with preconception hypoglycemia were found to have increased risks of LBW, SGA, and birth defects, contrasting with Zeng and colleagues’ findings using the same definition of hypoglycemia but a different reference group (FPG 3.9–6.0 mmol/L) [30]. Moreover, the effect of preconception hypoglycemia on LBW in our study is comparable to that of hypoglycemia identified during pregnancy with different sample sizes (*n* = 805, 625, and 3,537, respectively), oral glucose tolerance tests (100-g, 75-g, and 75-g, respectively), and definitions of hypoglycemia (blood glucose < 2.8, 3.9, and 3.5 mmol/L, respectively) [21,25,27]. Our findings indicated that women with preconception hypoglycemia might be at risk of some adverse pregnancy outcomes, emphasizing the importance of early detection and appropriate intervention in managing preconception glucose among childbearing-aged women to ensure a safe and healthy pregnancy.

Questions have been raised about the potential benefits of maternal preconception hypoglycemia in pregnancy outcomes. In our study, women with preconception hypoglycemia have decreased risks of medical abortion, miscarriage or early stillbirth, macrosomia, and LGA. However, the associations of hypoglycemia with macrosomia and LGA were inconsistent with Zeng and colleagues’ findings that maternal preconception hypoglycemia might increase the risk of macrosomia (Adjusted RR 1.17, 95% CI [0.98, 1.40]) and LGA (Adjusted RR 1.06, [0.94, 1.20]) [30]. As previously reported, women with lower preconception FPG levels (<3.9 mmol/L) had a decreased risk of infertility compared to those with normal FPG, which might link up with a lower risk of subsequent spontaneous abortion [46,47]. However, given the limited evidence, the effects of maternal preconception hypoglycemia on medical abortion, miscarriage or early stillbirth, macrosomia, and LGA should be interpreted cautiously.

Hypoglycemia is a complex and multifactorial condition resulting from disruptions in the intricate regulatory mechanisms that govern glucose homeostasis. There are several possible pathogeneses that cause endogenous hyperinsulinemia and subsequently result in hypoglycemia, including insulinoma, non-insulinoma pancreatogenesis hypoglycemia syndrome, and autoimmune hypoglycemia syndrome [48]. Moreover, hormone deficiency, like adrenal, might also be a possible pathogenesis resulting in hypoglycemia. Since cortisol exerts critical metabolic effects by impairing insulin signaling, increasing gluconeogenesis, lipolysis, ketogenesis, and proteolysis, and decreasing glucose utilization, its deficiency might impair counter-regulatory defenses against hypoglycemia [48]. Participants who have preconception hypoglycemia might require referral for a more comprehensive examination to ascertain the detailed cause and appropriate medical treatment before they get pregnant to reduce adverse pregnancy outcomes.

In addition, specific estimations of the associations according to preconception BMI were performed to identify potential strategies regarding reducing adverse pregnancy outcomes for those women with preconception hypoglycemia. Findings from modification analyses indicated that women with preconception hypoglycemia might benefit from achieving a suitable BMI and maintaining it throughout pregnancy once hypoglycemia cannot be corrected promptly, as maternal preconception BMI and gestational weight gain have been reported to be related to adverse pregnancy outcomes [49–51]. However, further prospective studies with detailed information on gestational weight gain are necessary to provide more comprehensive strategies for women with preconception hypoglycemia to reduce adverse pregnancy outcomes.

The main strength of this study is the use of a nationwide, population-based project, which enables us to have nationally representative estimates of the impact of preconception hypoglycemia on adverse pregnancy outcomes among women with preconception FPG < 5.6 mmol/L. In addition, our study included detailed demographic and clinical information regarding lifestyle, previous adverse pregnancy outcomes, current disease status, and other key confounding variables in the analyses. Indeed, associations are independent of all known risk factors of adverse pregnancy outcomes, even after restricting the analysis to participants without a history of adverse pregnancy outcomes or pre-existing diabetes. The study provides comprehensive evidence of associations between maternal preconception hypoglycemia and adverse pregnancy outcomes, with consideration of the modification effect of preconception BMI, which might be worth exploring to reduce adverse pregnancy outcomes.

However, some limitations in our study should be mentioned. Firstly, this is a retrospective study, and we only included Chinese women; further prospective studies in other populations are essential. Secondly, FPG was measured only once, and HbA1c was not measured; hence, the transient hypoglycemia status could not be excluded. Also, based on the FPG results, women with hypoglycemia might be referred to medical treatment; this might affect the pregnancy and the outcome measurement. Thirdly, due to the limitations of the survey and full clinical examinations, not all confounders were included. When considering the impact of unmeasured confounding through computed *E*-values, we found that, in contrast to the associations concerning PTB, macrosomia, LGA, and birth defects, which are unlikely to be offset by an unobserved confounder, the reported associations for medical abortion (*E*-values = 1.32), miscarriage or early stillbirth (*E*-value = 1.29), LBW (*E*-value = 1.37), SGA (*E*-value = 1.34), and perinatal death (*E*-value = 1.24), respectively. Fourth, as early pregnancy information and pregnancy outcomes were collected via telephone by trained healthcare, there was no exact time available for the occurrence of some pregnancy outcomes, such as ectopic pregnancy and medical terminations. Thus, no competing risk model or inverse probability of censoring weights could be performed. Finally, as the goal of NFPCP project was to identify the potential risk factors prior to pregnancy that could increase the risk of adverse pregnancy outcomes, no detailed and accurate information on maternal gestational weight gain and gestational complications, such as gestational diabetes and hypertensive disorders in pregnancy, was collected. Further longitudinal and high-quality studies are needed to confirm the effect of preconception hypoglycemia on both gestational complications and pregnancy outcomes.

In summary, maternal preconception hypoglycemia is significantly associated with various adverse pregnancy outcomes, and maternal preconception BMI could modify the impact of preconception hypoglycemia. Our findings indicated that further attention should be paid to women with hypoglycemia prior to pregnancy, and screening for preconception hypoglycemia might be worth exploring as a means to reduce adverse pregnancy outcomes. Participants who have preconception hypoglycemia might require referral for a more comprehensive examination to ascertain the detailed cause and appropriate medical treatment before they get pregnant to reduce adverse pregnancy outcomes. Moreover, further study is warranted to determine whether improving nutritional status for women with preconception hypoglycemia reduces the risk of subsequent adverse pregnancy outcomes.

## Supporting information

S1 STROBE Checklist Abbreviation: STROBE, Strengthening the Reporting of Observational Studies in Epidemiology. (DOCX)

S1 Fig Directed acyclic graph for the association between maternal preconception hypoglycemia (exposure) and risks of adverse pregnancy outcomes (outcome), incorporating causal pathways and covariates. (TIFF)

S2 Fig Dose-response relationship between maternal preconception fasting plasma glucose and risk of various adverse pregnancy outcomes (unweighted-multivariate adjusted models). The graph shows the unweighted-multivariate adjusted OR of association between maternal preconception FPG and the risk of adverse pregnancy outcomes. In the graph, black curves and shaded gray areas show predicted OR and 95% CI, respectively. Abbreviation: OR, odds ratio; CI, confidence interval.
(TIFF)

S1 Table Definition and classification of covariates. Abbreviation: GDP, gross domestic product; BMI, body mass index; CNY, Chinese Yuan.
(DOCX)

S2 Table Sensitivity analysis of the association between preconception hypoglycemia and adverse pregnancy outcomes after excluding participants with a history of adverse pregnancy outcomes. Model was adjusted for maternal age, ethnicity, educational level, occupation, region, smoking, passive smoking, alcohol consumption, maternal preconception BMI, parity, preconception medicine use, folic acid use, hypertension, diabetes, anemia, thyroid disorder, liver disorder, and infection. Abbreviations: FPG, fasting plasma glucose; CI, confidence interval; OR, odds ratio; BMI, body mass index; IPTW, inverse probability treatment weighting.
(DOCX)

S3 Table Sensitivity analysis of the association between preconception hypoglycemia and adverse pregnancy outcomes after excluding participants with pre-existing diabetes. Model was adjusted with maternal age, ethnicity, educational level, occupation, region, smoking, passive smoking, alcohol consumption, maternal preconception BMI, parity, preconception medicine use, folic acid use, hypertension, anemia, thyroid disorder, liver disorder, and infection. Abbreviations: FPG, fasting plasma glucose; CI, confidence interval; OR, odds ratio; BMI, body mass index; IPTW, inverse probability treatment weighting.
(DOCX)

S4 Table Association between preconception hypoglycemia and adverse pregnancy outcomes stratified by maternal preconception BMI status. Underweight, BMI < 18.5 kg/m^2^; Normal weight, BMI between 18.5 and 23.9 kg/m^2^; Overweight, BMI between 24.0 and 27.9 kg/m^2^; Obesity, BMI ≥ 28.0 kg/m^2^. Model was adjusted for maternal age, ethnicity, educational level, occupation, region, smoking, passive smoking, alcohol consumption, parity, preconception medicine use, folic acid use, hypertension, diabetes, anemia, thyroid disorder, liver disorder, and infection. Abbreviations: IPTW, inverse probability treatment weighting; OR, odds ratio; CI, confidence interval; BMI, body mass index.
(DOCX)

S5 Table Modification effect of maternal preconception BMI on the association between preconception hypoglycemia and adverse pregnancy outcomes. Underweight, BMI < 18.5 kg/m^2^; Normal weight, BMI between 18.5 and 23.9 kg/m^2^; Overweight, BMI between 24.0 and 27.9 kg/m^2^; Obesity, BMI ≥ 28.0 kg/m^2^. Model was adjusted for maternal age, ethnicity, educational level, occupation, region, smoking, passive smoking, alcohol consumption, parity, preconception medicine use, folic acid use, hypertension, diabetes, anemia, thyroid disorder, liver disorder, and infection. Abbreviations: IPTW, inverse probability treatment weighting; FPG, fasting plasma glucose; RERI, relative excess risk due to interaction; OR, odds ratio; CI, confidence interval; BMI, body mass index.
(DOCX)

## Abbreviations

ADA: American Diabetes Association
BMI: body mass index
CIs: confidence intervals
CNY: Chinese yuan
DAG: directed acyclic graph
FDR: false discovery rate
FPG: fasting plasma glucose
GDP: gross domestic product
IPTW: inverse probability treatment weighting
IQRs: interquartile ranges
LBW: low birth weight
LGA: large for gestational age
MSAS: minimal sufficient adjustment set
NFPCP: National Free Preconception Checkups Project
ORs: odds ratios
PTB: preterm birth
RCS: restricted cubic spline
RERI: relative excess risk due to interaction
SGA: small for gestational age
SMD: standardized mean difference
STROBE: Strengthening the Reporting of Observational Studies in Epidemiology
