# Supplementary material for: Preconception hypoglycemia and adverse pregnancy outcomes in Chinese women aged 20–49 years: A retrospective cohort study in China
Source: PLoS Med. 2025 Jul 29;22(7):e1004667. doi: 10.1371/journal.pmed.1004667 (PMC12306775; doi:10.1371/journal.pmed.1004667)
Supplement: S1 STROBE Checklist — (DOCX) [file pmed.1004667.s001.docx]

STROBE Statement—Checklist of items that should be included in reports of ***cohort studies***

|  | Item No | Recommendation | Page |
| --- | --- | --- | --- |
| **Title and abstract** | 1 | (*a*) Indicate the study’s design with a commonly used term in the title or the abstract | Title section on page 1, lines 2-3.  Abstract section on pages 4-6, lines 62-113. |
|  |  | (*b*) Provide in the abstract an informative and balanced summary of what was done and what was found | Abstract section on pages 4-6, lines 62-113 |
| Introduction | | |  |
| Background/rationale | 2 | Explain the scientific background and rationale for the investigation being reported | Introduction section on pages 9-10, lines 153-183. |
| Objectives | 3 | State specific objectives, including any prespecified hypotheses | Introduction section on page 10, lines 184-187. |
| Methods | | |  |
| Study design | 4 | Present key elements of study design early in the paper | Method section on page 11, lines 191-193. |
| Setting | 5 | Describe the setting, locations, and relevant dates, including periods of recruitment, exposure, follow-up, and data collection | Method section on pages 11-13, lines 193-245. |
| Participants | 6 | (*a*) Give the eligibility criteria, and the sources and methods of selection of participants. Describe methods of follow-up | Method section on pages 11-13, lines 197-245. |
|  |  | (*b*) For matched studies, give matching criteria and number of exposed and unexposed | N/A |
| Variables | 7 | Clearly define all outcomes, exposures, predictors, potential confounders, and effect modifiers. Give diagnostic criteria, if applicable | Method section on pages 13-14, lines 246-288. |
| Data sources/ measurement | 8* | For each variable of interest, give sources of data and details of methods of assessment (measurement). Describe comparability of assessment methods if there is more than one group | Method section on pages 13-14, lines 246-267. |
| Bias | 9 | Describe any efforts to address potential sources of bias | Method section on pages 15-16, lines 290-301, and 306-331. |
| Study size | 10 | Explain how the study size was arrived at | Method section on pages 12-13, lines 234-243. |
| Quantitative variables | 11 | Explain how quantitative variables were handled in the analyses. If applicable, describe which groupings were chosen and why | Method section on pages 13-14, lines 246-253 and 268-288. |
| Statistical methods | 12 | (*a*) Describe all statistical methods, including those used to control for confounding | Method section on pages 15-16, lines 290-336. |
|  |  | (*b*) Describe any methods used to examine subgroups and interactions | Method section on page 16, lines 320-324. |
|  |  | (*c*) Explain how missing data were addressed | Method section on page 14, lines 286-288. |
|  |  | (*d*) If applicable, explain how loss to follow-up was addressed | N/A |
|  |  | (*e*) Describe any sensitivity analyses | Method section on page 16, lines 325-331. |
| Results | | |  |
| Participants | 13* | (a) Report numbers of individuals at each stage of study—eg numbers potentially eligible, examined for eligibility, confirmed eligible, included in the study, completing follow-up, and analysed | Result section on page 17, lines 341-347. |
|  |  | (b) Give reasons for non-participation at each stage | N/A |
|  |  | (c) Consider use of a flow diagram | Figure 2 |
| Descriptive data | 14* | (a) Give characteristics of study participants (eg demographic, clinical, social) and information on exposures and potential confounders | Result section on page 17, lines 341-347, and Table 1 on pages 18-19, lines 349-351. |
|  |  | (b) Indicate number of participants with missing data for each variable of interest | Table 1 on pages 18-19, lines 349-351. |
|  |  | (c) Summarise follow-up time (eg, average and total amount) | Result section on page 17, lines 341-343. |
| Outcome data | 15* | Report numbers of outcome events or summary measures over time | Result section on page 20, lines 356-357, and Table 2 on pages 22-23, lines 379-383. |
| Main results | 16 | (*a*) Give unadjusted estimates and, if applicable, confounder-adjusted estimates and their precision (eg, 95% confidence interval). Make clear which confounders were adjusted for and why they were included | Result section on pages 20-21, lines 356-377, and Table 2 on pages 22-23, lines 379-383. |
|  |  | (*b*) Report category boundaries when continuous variables were categorized | N/A |
|  |  | (*c*) If relevant, consider translating estimates of relative risk into absolute risk for a meaningful time period | N/A |
| Other analyses | 17 | Report other analyses done—eg analyses of subgroups and interactions, and sensitivity analyses | Result section on pages 24-26, lines 387-437. |
| Discussion | | |  |
| Key results | 18 | Summarise key results with reference to study objectives | Discussion section on page 27, lines 440-449. |
| Limitations | 19 | Discuss limitations of the study, taking into account sources of potential bias or imprecision. Discuss both direction and magnitude of any potential bias | Discussion section on pages 30-31, lines 521-542. |
| Interpretation | 20 | Give a cautious overall interpretation of results considering objectives, limitations, multiplicity of analyses, results from similar studies, and other relevant evidence | Discussion section on pages 27-29, lines 450-509. |
| Generalisability | 21 | Discuss the generalisability (external validity) of the study results | Discussion section on page 31, lines 543-552. |
| Other information | | |  |
| Funding | 22 | Give the source of funding and the role of the funders for the present study and, if applicable, for the original study on which the present article is based | Title section on page 2, lines 42-50.  Funding section on pages 32-33, lines 574-582. |

*Give information separately for exposed and unexposed groups.

**Note:** An Explanation and Elaboration article discusses each checklist item and gives methodological background and published examples of transparent reporting. The STROBE checklist is best used in conjunction with this article (freely available on the Web sites of PLoS Medicine at http://www.plosmedicine.org/, Annals of Internal Medicine at http://www.annals.org/, and Epidemiology at http://www.epidem.com/). Information on the STROBE Initiative is available at http://www.strobe-statement.org.
