# Supplementary figures and images for: Preconception hypoglycemia and adverse pregnancy outcomes in Chinese women aged 20–49 years: A retrospective cohort study in China
Source: PLoS Med. 2025 Jul 29;22(7):e1004667. doi: 10.1371/journal.pmed.1004667 (PMC12306775; doi:10.1371/journal.pmed.1004667)

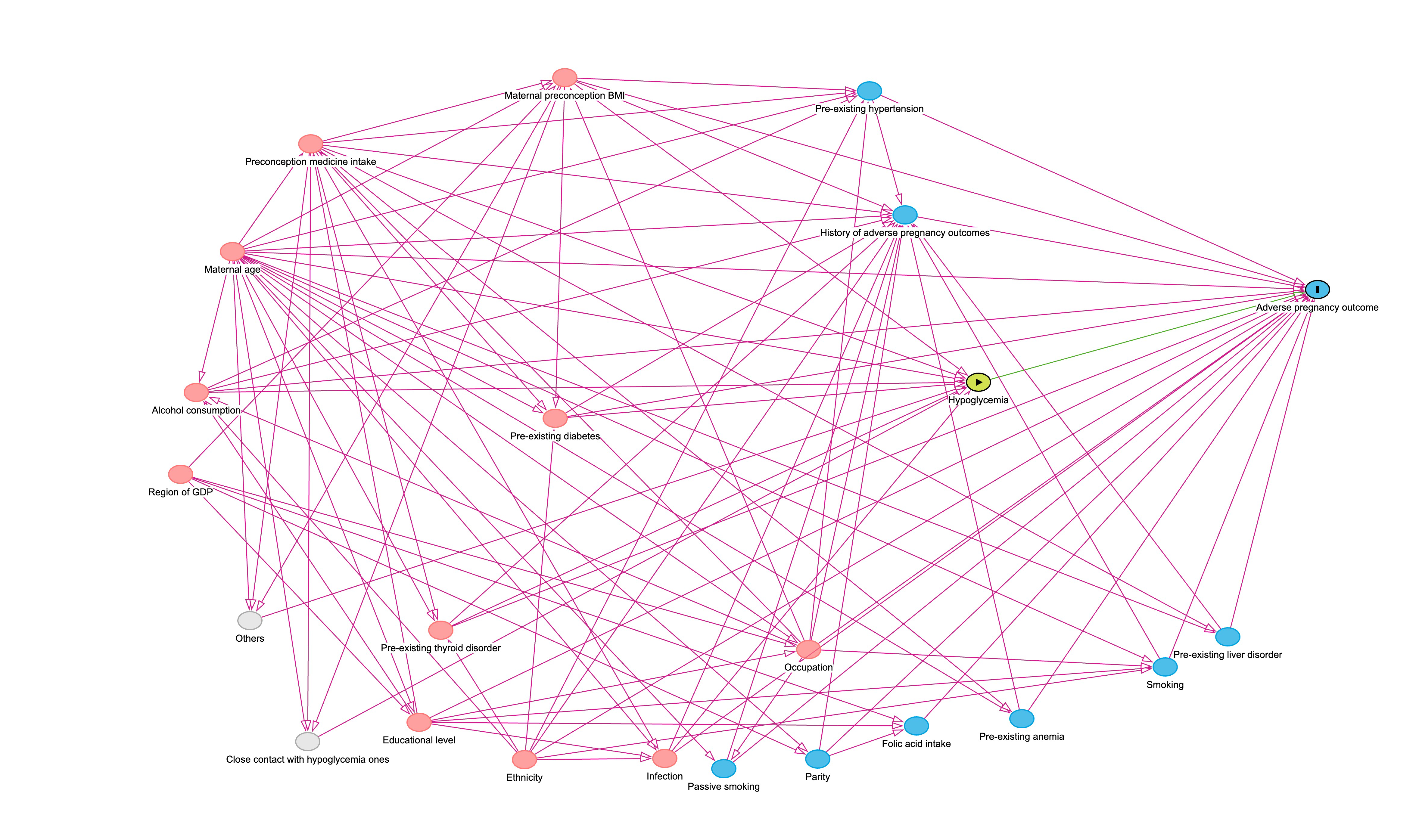

Supplement: S1 Fig — (TIFF) [file pmed.1004667.s002.tiff]

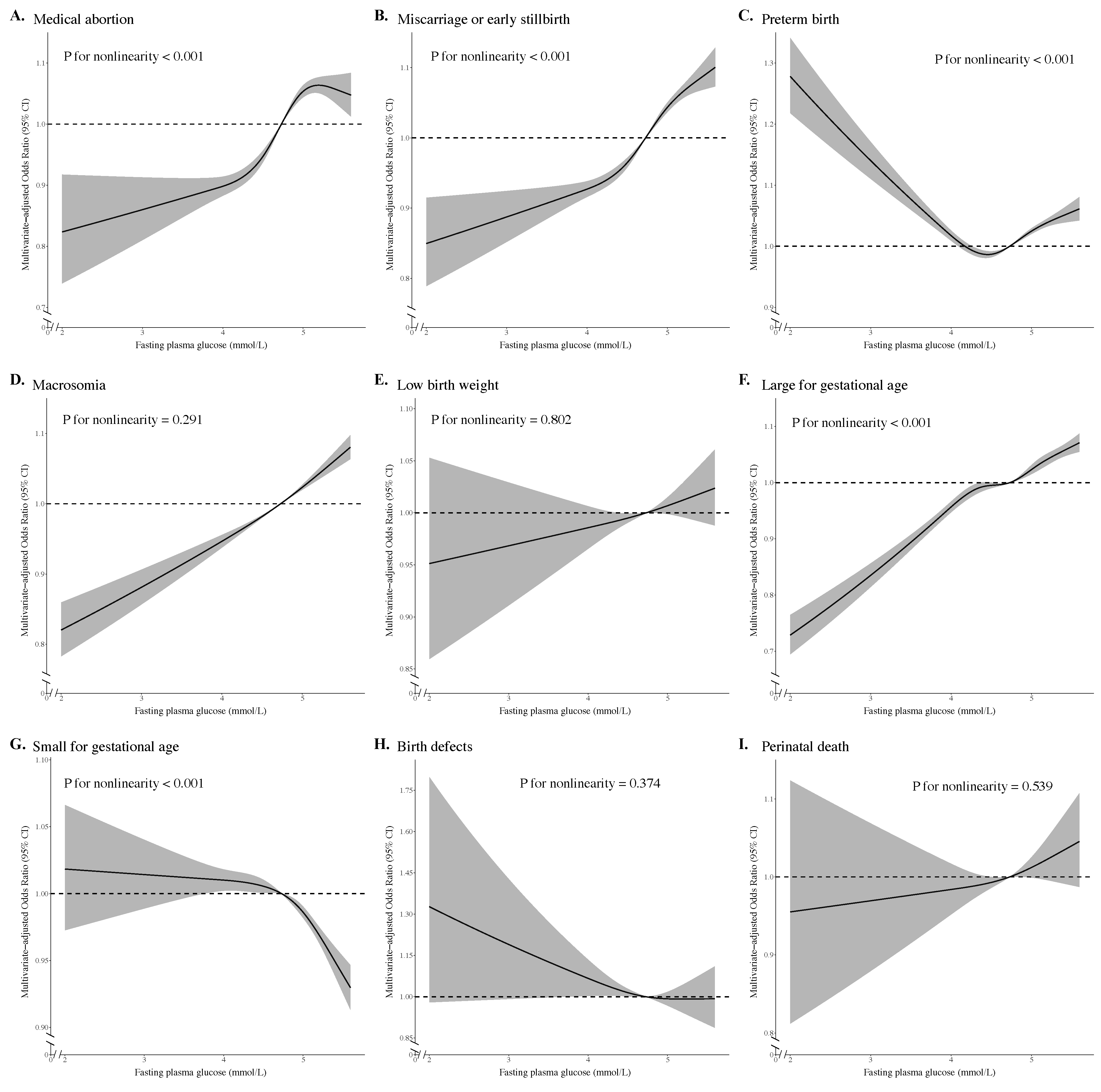

Supplement: S2 Fig — The graph shows the unweighted-multivariate adjusted OR of association between maternal preconception FPG and the risk of adverse pregnancy outcomes. In the graph, black curves and shaded gray areas show predicted OR and 95% CI, respectively. Abbreviation: OR, odds ratio; CI, confidence interval. (TIFF) [file pmed.1004667.s003.tiff]
