# Supplementary material for: Preconception hypoglycemia and adverse pregnancy outcomes in Chinese women aged 20–49 years: A retrospective cohort study in China
Source: PLoS Med. 2025 Jul 29;22(7):e1004667. doi: 10.1371/journal.pmed.1004667 (PMC12306775; doi:10.1371/journal.pmed.1004667)
Supplement: S1 Table — Abbreviation: GDP, gross domestic product; BMI, body mass index; CNY, Chinese Yuan. (DOCX) [file pmed.1004667.s004.docx]

**S1 Table. Definition and classification of covariates.**

| **Covariates** | **Definition** |
| --- | --- |
| **Demographic and sociological covariates:** | |
| Maternal age | Calculated by the date of last menstrual period minus the date of birth |
| Ethnicity | Self-reported (Han; others) |
| Education | Self-reported (illiterate, primary school, junior high school, senior high school, junior college, undergraduate and (or) postgraduate) |
| Occupation | Self-reported (farmer; non-farmer) |
| Region of GDP per capita | Calculated by the average real GDP per capita (≤ 40,000; 40,001-50,000; 50,001-70,000; > 70,000 CNY/year) |
| Smoking | Self-reported (Yes; no) |
| Passive smoking | Self-reported (Yes; no) |
| Alcohol consumption | Self-reported (Yes; no) |
| **Preconception clinical covariates:** | |
| BMI | Calculated by dividing the weight in kilogram by the square of the height in meters (underweight, <18.5 kg/m^2^; normal, 18.5-23.9 kg/m^2^; overweight, 24-27.9 kg/m^2^; obesity, ≥28.0 kg/m^2^) |
| Parity | Whether have previous live birth or stillbirth or not (Nulliparous; multiparous) |
| History of adverse pregnancy outcome | Self-reported, including spontaneous abortion, induction abortion, birth defect, or preterm birth (Yes; no) |
| Preconception medicine use | Self-reported (Yes; no) |
| Folic acid intake | Self-reported (Yes; no) |
| Hypertension | Self-reported history of hypertension or systolic blood pressure ≥ 140 mm Hg, and (or) diastolic blood pressure ≥ 90 mm Hg (Yes; no) |
| Diabetes | Self-reported history of diabetes (Yes; no) |
| Anemia | Self-reported history of anemia or serum hemoglobin ＜110 g/L (Yes; no) |
| Thyroid disorder | Self-reported history of thyroid disease, or serum thyroid stimulating hormone levels < 0.37 or > 4.88 μIU/ml |
| Liver disorder | Self-reported history of hepatitis, or serum alanine aminotransferase > 40U/L (Yes; no) |
| Infection | Women infected with Neisseria gonorrhoeae, Chlamydia trachomatis, Toxoplasma gondii, Cytomegalovirus, Treponema pallidum, hepatitis B virus, or Rubella virus, by detecting their specific-serum antibodies. (Yes; no) |

Abbreviation: GDP, gross domestic product; BMI, body mass index; CNY, Chinese Yuan.
