# Supplementary material for: Preconception hypoglycemia and adverse pregnancy outcomes in Chinese women aged 20–49 years: A retrospective cohort study in China
Source: PLoS Med. 2025 Jul 29;22(7):e1004667. doi: 10.1371/journal.pmed.1004667 (PMC12306775; doi:10.1371/journal.pmed.1004667)
Supplement: S2 Table — Model was adjusted for maternal age, ethnicity, educational level, occupation, region, smoking, passive smoking, alcohol consumption, maternal preconception BMI, parity, preconception medicine use, folic acid use, hypertension, diabetes, anemia, thyroid disorder, liver disorder, and infection. Abbreviations: FPG, fasting plasma glucose; CI, confidence interval; OR, odds ratio; BMI, body mass index; IPTW, inverse probability treatment weighting. (DOCX) [file pmed.1004667.s005.docx]

**S2 Table. Sensitivity analysis of the association between preconception hypoglycemia and adverse pregnancy outcomes after excluding participants with a history of adverse pregnancy outcomes.**

| **Outcomes** | **Cases/Participants (%)** | **Unweighted** | | | | **IPTW** | | | |
| --- | --- | --- | --- | --- | --- | --- | --- | --- | --- |
|  |  | **Unadjusted model** | | **Full model** | | **Unadjusted model** | | **Full model** | |
|  |  | **OR (95% CI)** | **P value** | **OR (95% CI)** | **P value** | **OR (95% CI)** | **P value** | **OR (95% CI)** | **P value** |
| **Medical abortion** |  |  |  |  |  |  |  |  |  |
| 3.9- <5.6 mmol/L | 61,689/4,474,580 (1.38%) | 1.00 Reference | ··· | 1.00 Reference | ··· | 1.00 Reference | ··· | 1.00 Reference | ··· |
| < 3.9 mmol/L | 2,780/231,701 (1.20%) | 0.87 (0.84, 0.90) | <0.001 | 0.94 (0.91, 0.98) | 0.004 | 0.88 (0.84, 0.91) | <0.001 | 0.95 (0.91, 0.98) | 0.005 |
| **Miscarriage or early stillbirth** |  |  |  |  |  |  |  |  |  |
| 3.9- <5.6 mmol/L | 122,618/4,474,580 (2.74%) | 1.00 Reference | ··· | 1.00 Reference | ··· | 1.00 Reference | ··· | 1.00 Reference | ··· |
| < 3.9 mmol/L | 5,939/231,701 (2.56%) | 0.93 (0.91, 0.96) | <0.001 | 0.93 (0.90, 0.95) | <0.001 | 0.94 (0.91, 0.96) | <0.001 | 0.93 (0.90, 0.95) | <0.001 |
| **Preterm birth** |  |  |  |  |  |  |  |  |  |
| 3.9- <5.6 mmol/L | 259,012/4,153,677 (6.24%) | 1.00 Reference | ··· | 1.00 Reference | ··· | 1.00 Reference | ··· | 1.00 Reference | ··· |
| < 3.9 mmol/L | 14,868/214,804 (6.92%) | 1.12 (1.10, 1.14) | <0.001 | 1.11 (1.09, 1.13) | <0.001 | 1.12 (1.10, 1.14) | <0.001 | 1.11 (1.09, 1.13) | <0.001 |
| **Macrosomia** |  |  |  |  |  |  |  |  |  |
| 3.9- <5.6 mmol/L | 210,214/4,142,889 (5.07%) | 1.00 Reference | ··· | 1.00 Reference | ··· | 1.00 Reference | ··· | 1.00 Reference | ··· |
| < 3.9 mmol/L | 9,077/214,325 (4.24%) | 0.83 (0.81, 0.85) | <0.001 | 0.88 (0.86, 0.90) | <0.001 | 0.83 (0.82, 0.85) | <0.001 | 0.88 (0.86, 0.90) | <0.001 |
| **Low birth weight** |  |  |  |  |  |  |  |  |  |
| 3.9- <5.6 mmol/L | 37,057/3,969,732 (0.93%) | 1.00 Reference | ··· | 1.00 Reference | ··· | 1.00 Reference | ··· | 1.00 Reference | ··· |
| < 3.9 mmol/L | 2,142/207,390 (1.03%) | 1.11 (1.06, 1.16) | <0.001 | 1.06 (1.02, 1.11) | 0.006 | 1.10 (1.06, 1.15) | <0.001 | 1.07 (1.02, 1.11) | 0.004 |
| **Large for gestational age** |  |  |  |  |  |  |  |  |  |
| 3.9- <5.6 mmol/L | 408,238/3,880,317 (10.52%) | 1.00 Reference | ··· | 1.00 Reference | ··· | 1.00 Reference | ··· | 1.00 Reference | ··· |
| < 3.9 mmol/L | 17,474/198,738 (8.79%) | 0.82 (0.81, 0.83) | <0.001 | 0.87 (0.86, 0.89) | <0.001 | 0.83 (0.81, 0.84) | <0.001 | 0.87 (0.86, 0.89) | <0.001 |
| **Small for gestational age** |  |  |  |  |  |  |  |  |  |
| 3.9- <5.6 mmol/L | 285,343/3,757,422 (7.59%) | 1.00 Reference | ··· | 1.00 Reference | ··· | 1.00 Reference | ··· | 1.00 Reference | ··· |
| < 3.9 mmol/L | 16,947/198,211 (8.55%) | 1.14 (1.12, 1.16) | <0.001 | 1.06 (1.04, 1.08) | <0.001 | 1.13 (1.11, 1.15) | <0.001 | 1.06 (1.04, 1.08) | <0.001 |
| **Birth defects** |  |  |  |  |  |  |  |  |  |
| 3.9- <5.6 mmol/L | 3,764/4,474,580 (0.08%) | 1.00 Reference | ··· | 1.00 Reference | ··· | 1.00 Reference | ··· | 1.00 Reference | ··· |
| < 3.9 mmol/L | 237/231,701 (0.10%) | 1.22 (1.07, 1.39) | 0.003 | 1.19 (1.04, 1.36) | 0.011 | 1.22 (1.07, 1.39) | 0.003 | 1.19 (1.05, 1.36) | 0.008 |
| **Perinatal death** |  |  |  |  |  |  |  |  |  |
| 3.9- <5.6 mmol/L | 14,574/4,474,580 (0.33%) | 1.00 Reference | ··· | 1.00 Reference | ··· | 1.00 Reference | ··· | 1.00 Reference | ··· |
| < 3.9 mmol/L | 803/231,701 (0.35%) | 1.06 (0.99, 1.14) | 0.086 | 1.03 (0.96, 1.11) | 0.436 | 1.06 (0.99, 1.14) | 0.107 | 1.03 (0.96, 1.10) | 0.435 |
|  |  |  |  |  |  |  |  |  |  |

Model was adjusted for maternal age, ethnicity, educational level, occupation, region, smoking, passive smoking, alcohol consumption, maternal preconception BMI, parity, preconception medicine use, folic acid use, hypertension, diabetes, anemia, thyroid disorder, liver disorder, and infection.

Abbreviations: IPTW, inverse probability of treatment weighted; OR, odds ratio; CI, confidence interval; BMI, body mass index.
