# Supplementary material for: Preconception hypoglycemia and adverse pregnancy outcomes in Chinese women aged 20–49 years: A retrospective cohort study in China
Source: PLoS Med. 2025 Jul 29;22(7):e1004667. doi: 10.1371/journal.pmed.1004667 (PMC12306775; doi:10.1371/journal.pmed.1004667)
Supplement: S4 Table — Underweight, BMI < 18.5 kg/m2; Normal weight, BMI between 18.5 and 23.9 kg/m2; Overweight, BMI between 24.0 and 27.9 kg/m2; Obesity, BMI ≥ 28.0 kg/m2. Model was adjusted for maternal age, ethnicity, educational level, occupation, region, smoking, passive smoking, alcohol consumption, parity, preconception medicine use, folic acid use, hypertension, diabetes, anemia, thyroid disorder, liver disorder, and infection. Abbreviations: IPTW, inverse probability treatment weighting; OR, odds ratio; CI, confidence interval; BMI, body mass index. (DOCX) [file pmed.1004667.s007.docx]

**S4 Table. Association between preconception hypoglycemia and adverse pregnancy outcomes stratified by maternal preconception BMI status.**

| **Outcome** | **Cases/Participants (%)** | **Unweighted** | | | | **IPTW** | | | |
| --- | --- | --- | --- | --- | --- | --- | --- | --- | --- |
|  |  | **Unadjusted model** | | **Full model** | | **Unadjusted model** | | **Full model** | |
|  |  | **OR (95% CI)** | **P value** | **OR (95% CI)** | **P value** | **OR (95% CI)** | **P value** | **OR (95% CI)** | **P value** |
| **Medical abortion** |  |  |  |  |  |  |  |  |  |
| Underweight |  |  |  |  |  |  |  |  |  |
|  | 8,448/627,851 (1.35%) | 1.00 Reference | ··· | 1.00 Reference | ··· | 1.00 Reference | ··· | 1.00 Reference | ··· |
|  | 446/41,784 (1.07%) | 0.79 (0.72, 0.87) | <0.001 | 0.83 (0.75, 0.91) | <0.001 | 0.80 (0.72, 0.88) | <0.001 | 0.83 (0.75, 0.91) | <0.001 |
| Normal |  |  |  |  |  |  |  |  |  |
|  | 44,125/3,311,635 (1.33%) | 1.00 Reference | ··· | 1.00 Reference | ··· | 1.00 Reference | ··· | 1.00 Reference | ··· |
|  | 2,028/169,723 (1.19%) | 0.90 (0.86, 0.94) | <0.001 | 0.96 (0.92, 1.01) | 0.105 | 0.90 (0.86, 0.94) | <0.001 | 0.96 (0.92, 1.01) | 0.109 |
| Overweight |  |  |  |  |  |  |  |  |  |
|  | 9,622/550,696 (1.75%) | 1.00 Reference | ··· | 1.00 Reference | ··· | 1.00 Reference | ··· | 1.00 Reference | ··· |
|  | 341/22,569 (1.51%) | 0.86 (0.77, 0.96) | 0.008 | 0.96 (0.86, 1.07) | 0.476 | 0.87 (0.78, 0.97) | 0.013 | 0.96 (0.86, 1.07) | 0.494 |
| Obesity |  |  |  |  |  |  |  |  |  |
|  | 2,350/120,306 (1.95%) | 1.00 Reference | ··· | 1.00 Reference | ··· | 1.00 Reference | ··· | 1.00 Reference | ··· |
|  | 78/4,303 (1.81%) | 0.93 (0.74, 1.16) | 0.512 | 1.03 (0.82, 1.29) | 0.828 | 0.94 (0.75, 1.17) | 0.561 | 1.03 (0.82, 1.29) | 0.813 |
| **Miscarriage or early stillbirth** |  |  |  |  |  |  |  |  |  |
| Underweight |  |  |  |  |  |  |  |  |  |
|  | 17,604/627,851 (2.80%) | 1.00 Reference | ··· | 1.00 Reference | ··· | 1.00 Reference | ··· | 1.00 Reference | ··· |
|  | 1,018/41,784 (2.44%) | 0.87 (0.81, 0.92) | <0.001 | 0.87 (0.82, 0.93) | <0.001 | 0.87 (0.81, 0.92) | <0.001 | 0.87 (0.82, 0.93) | <0.001 |
| Normal |  |  |  |  |  |  |  |  |  |
|  | 89,526/3,311,635 (2.70%) | 1.00 Reference | ··· | 1.00 Reference | ··· | 1.00 Reference | ··· | 1.00 Reference | ··· |
|  | 4,362/169,723 (2.57%) | 0.95 (0.92, 0.98) | 0.001 | 0.96 (0.93, 0.99) | 0.005 | 0.95 (0.92, 0.98) | 0.001 | 0.96 (0.93, 0.99) | 0.006 |
| Overweight |  |  |  |  |  |  |  |  |  |
|  | 17,715/550,696 (3.22%) | 1.00 Reference | ··· | 1.00 Reference | ··· | 1.00 Reference | ··· | 1.00 Reference | ··· |
|  | 715/22,569 (3.17%) | 0.98 (0.91, 1.06) | 0.684 | 0.95 (0.88, 1.03) | 0.237 | 0.98 (0.91, 1.06) | 0.638 | 0.95 (0.88, 1.03) | 0.236 |
| Obesity |  |  |  |  |  |  |  |  |  |
|  | 4,129/120,306 (3.43%) | 1.00 Reference | ··· | 1.00 Reference | ··· | 1.00 Reference | ··· | 1.00 Reference | ··· |
|  | 165/4,303 (3.83%) | 1.12 (0.96, 1.31) | 0.155 | 1.08 (0.92, 1.26) | 0.370 | 1.12 (0.96, 1.31) | 0.154 | 1.08 (0.92, 1.27) | 0.353 |
| **Preterm birth** |  |  |  |  |  |  |  |  |  |
| Underweight |  |  |  |  |  |  |  |  |  |
|  | 38,152/582,069 (6.55%) | 1.00 Reference | ··· | 1.00 Reference | ··· | 1.00 Reference | ··· | 1.00 Reference | ··· |
|  | 2,660/38,933 (6.83%) | 1.05 (1.00, 1.09) | 0.032 | 1.03 (0.99, 1.08) | 0.127 | 1.04 (1.00, 1.09) | 0.044 | 1.03 (0.99, 1.07) | 0.136 |
| Normal |  |  |  |  |  |  |  |  |  |
|  | 189,076/3,075,572 (6.15%) | 1.00 Reference | ··· | 1.00 Reference | ··· | 1.00 Reference | ··· | 1.00 Reference | ··· |
|  | 10,858/157,247 (6.91%) | 1.13 (1.11, 1.16) | <0.001 | 1.11 (1.09, 1.14) | <0.001 | 1.13 (1.11, 1.15) | <0.001 | 1.11 (1.09, 1.14) | <0.001 |
| Overweight |  |  |  |  |  |  |  |  |  |
|  | 33,566/508,342 (6.60%) | 1.00 Reference | ··· | 1.00 Reference | ··· | 1.00 Reference | ··· | 1.00 Reference | ··· |
|  | 1,565/20,718 (7.55%) | 1.16 (1.10, 1.22) | <0.001 | 1.15 (1.09, 1.21) | <0.001 | 1.15 (1.09, 1.21) | <0.001 | 1.15 (1.09, 1.21) | <0.001 |
| Obesity |  |  |  |  |  |  |  |  |  |
|  | 7,747/110,759 (6.99%) | 1.00 Reference | ··· | 1.00 Reference | ··· | 1.00 Reference | ··· | 1.00 Reference | ··· |
|  | 298/3,916 (7.61%) | 1.10 (0.97, 1.24) | 0.139 | 1.10 (0.98, 1.25) | 0.108 | 1.09 (0.97, 1.23) | 0.147 | 1.10 (0.98, 1.24) | 0.113 |
| **Macrosomia** |  |  |  |  |  |  |  |  |  |
| Underweight |  |  |  |  |  |  |  |  |  |
|  | 20,686/578,414 (3.58%) | 1.00 Reference | ··· | 1.00 Reference | ··· | 1.00 Reference | ··· | 1.00 Reference | ··· |
|  | 1,259/38,724 (3.25%) | 0.91 (0.86, 0.96) | 0.001 | 0.94 (0.88, 0.99) | 0.030 | 0.91 (0.86, 0.96) | 0.001 | 0.94 (0.88, 0.99) | 0.024 |
| Normal |  |  |  |  |  |  |  |  |  |
|  | 153,073/3,068,827 (4.99%) | 1.00 Reference | ··· | 1.00 Reference | ··· | 1.00 Reference | ··· | 1.00 Reference | ··· |
|  | 6,649/156,976 (4.24%) | 0.84 (0.82, 0.86) | <0.001 | 0.88 (0.86, 0.90) | <0.001 | 0.85 (0.82, 0.87) | <0.001 | 0.88 (0.85, 0.90) | <0.001 |
| Overweight |  |  |  |  |  |  |  |  |  |
|  | 34,364/507,084 (6.78%) | 1.00 Reference | ··· | 1.00 Reference | ··· | 1.00 Reference | ··· | 1.00 Reference | ··· |
|  | 1,181/20,677 (5.71%) | 0.83 (0.78, 0.88) | <0.001 | 0.88 (0.83, 0.93) | <0.001 | 0.84 (0.79, 0.89) | <0.001 | 0.88 (0.83, 0.93) | <0.001 |
| Obesity |  |  |  |  |  |  |  |  |  |
|  | 8,551/110,397 (7.75%) | 1.00 Reference | ··· | 1.00 Reference | ··· | 1.00 Reference | ··· | 1.00 Reference | ··· |
|  | 251/3,917 (6.41%) | 0.82 (0.72, 0.93) | 0.002 | 0.86 (0.76, 0.98) | 0.025 | 0.82 (0.72, 0.93) | 0.002 | 0.86 (0.76, 0.98) | 0.024 |
| **Low birth weight** |  |  |  |  |  |  |  |  |  |
| Underweight |  |  |  |  |  |  |  |  |  |
|  | 7,556/565,284 (1.34%) | 1.00 Reference | ··· | 1.00 Reference | ··· | 1.00 Reference | ··· | 1.00 Reference | ··· |
|  | 501/37,966 (1.32%) | 0.99 (0.90, 1.08) | 0.779 | 0.99 (0.91, 1.09) | 0.888 | 0.99 (0.90, 1.08) | 0.792 | 0.99 (0.91, 1.09) | 0.912 |
| Normal |  |  |  |  |  |  |  |  |  |
|  | 25,863/2,941,617 (0.88%) | 1.00 Reference | ··· | 1.00 Reference | ··· | 1.00 Reference | ··· | 1.00 Reference | ··· |
|  | 1,486/151,813 (0.98%) | 1.11 (1.06, 1.17) | <0.001 | 1.10 (1.04, 1.16) | 0.001 | 1.11 (1.06, 1.17) | 0.000 | 1.10 (1.04, 1.16) | <0.001 |
| Overweight |  |  |  |  |  |  |  |  |  |
|  | 4,772/477,492 (1.00%) | 1.00 Reference | ··· | 1.00 Reference | ··· | 1.00 Reference | ··· | 1.00 Reference | ··· |
|  | 220/19,716 (1.12%) | 1.12 (0.98, 1.28) | 0.108 | 1.13 (0.99, 1.30) | 0.078 | 1.12 (0.98, 1.28) | 0.107 | 1.13 (0.99, 1.29) | 0.078 |
| Obesity |  |  |  |  |  |  |  |  |  |
|  | 1,189/103,035 (1.15%) | 1.00 Reference | ··· | 1.00 Reference | ··· | 1.00 Reference | ··· | 1.00 Reference | ··· |
|  | 46/3,712 (1.24%) | 1.07 (0.80, 1.45) | 0.633 | 1.09 (0.81, 1.46) | 0.588 | 1.08 (0.80, 1.45) | 0.618 | 1.09 (0.81, 1.46) | 0.568 |
| **Large for gestational age** |  |  |  |  |  |  |  |  |  |
| Underweight |  |  |  |  |  |  |  |  |  |
|  | 41,626/526,461 (7.91%) | 1.00 Reference | ··· | 1.00 Reference | ··· | 1.00 Reference | ··· | 1.00 Reference | ··· |
|  | 2,483/34,979 (7.10%) | 0.89 (0.85, 0.93) | <0.001 | 0.92 (0.89, 0.96) | <0.001 | 0.89 (0.86, 0.93) | <0.001 | 0.92 (0.88, 0.96) | <0.001 |
| Normal |  |  |  |  |  |  |  |  |  |
|  | 298,443/2,879,439 (10.36%) | 1.00 Reference | ··· | 1.00 Reference | ··· | 1.00 Reference | ··· | 1.00 Reference | ··· |
|  | 12,898/146,049 (8.83%) | 0.84 (0.82, 0.85) | <0.001 | 0.88 (0.86, 0.89) | <0.001 | 0.84 (0.83, 0.86) | <0.001 | 0.87 (0.86, 0.89) | <0.001 |
| Overweight |  |  |  |  |  |  |  |  |  |
|  | 64,712/482,278 (13.42%) | 1.00 Reference | ··· | 1.00 Reference | ··· | 1.00 Reference | ··· | 1.00 Reference | ··· |
|  | 2,155/19,479 (11.06%) | 0.80 (0.77, 0.84) | <0.001 | 0.85 (0.81, 0.89) | <0.001 | 0.81 (0.77, 0.84) | <0.001 | 0.85 (0.81, 0.89) | <0.001 |
| Obesity |  |  |  |  |  |  |  |  |  |
|  | 15,563/105,300 (14.78%) | 1.00 Reference | ··· | 1.00 Reference | ··· | 1.00 Reference | ··· | 1.00 Reference | ··· |
|  | 444/3,686 (12.05%) | 0.79 (0.71, 0.87) | <0.001 | 0.84 (0.76, 0.93) | 0.001 | 0.79 (0.72, 0.88) | <0.001 | 0.84 (0.76, 0.93) | 0.001 |
| **Small for gestational age** |  |  |  |  |  |  |  |  |  |
| Underweight |  |  |  |  |  |  |  |  |  |
|  | 57,306/542,141 (10.57%) | 1.00 Reference | ··· | 1.00 Reference | ··· | 1.00 Reference | ··· | 1.00 Reference | ··· |
|  | 4,106/36,602 (11.22%) | 1.07 (1.03, 1.11) | <0.001 | 1.05 (1.02, 1.09) | 0.002 | 1.07 (1.03, 1.11) | <0.001 | 1.06 (1.02, 1.09) | 0.001 |
| Normal |  |  |  |  |  |  |  |  |  |
|  | 205,042/2,786,038 (7.36%) | 1.00 Reference | ··· | 1.00 Reference | ··· | 1.00 Reference | ··· | 1.00 Reference | ··· |
|  | 11,840/144,991 (8.17%) | 1.12 (1.10, 1.14) | <0.001 | 1.06 (1.04, 1.08) | <0.001 | 1.11 (1.09, 1.14) | <0.001 | 1.06 (1.04, 1.08) | <0.001 |
| Overweight |  |  |  |  |  |  |  |  |  |
|  | 27,461/445,027 (6.17%) | 1.00 Reference | ··· | 1.00 Reference | ··· | 1.00 Reference | ··· | 1.00 Reference | ··· |
|  | 1,327/18,651 (7.11%) | 1.16 (1.10, 1.23) | <0.001 | 1.09 (1.03, 1.16) | 0.002 | 1.16 (1.10, 1.23) | <0.001 | 1.10 (1.04, 1.16) | 0.002 |
| Obesity |  |  |  |  |  |  |  |  |  |
|  | 5,753/95,490 (6.02%) | 1.00 Reference | ··· | 1.00 Reference | ··· | 1.00 Reference | ··· | 1.00 Reference | ··· |
|  | 253/3,495 (7.24%) | 1.22 (1.07, 1.39) | 0.003 | 1.14 (1.00, 1.30) | 0.047 | 1.22 (1.07, 1.39) | 0.003 | 1.15 (1.01, 1.31) | 0.040 |
| **Birth defects** |  |  |  |  |  |  |  |  |  |
| Underweight |  |  |  |  |  |  |  |  |  |
|  | 602/627,851 (0.10%) | 1.00 Reference | ··· | 1.00 Reference | ··· | 1.00 Reference | ··· | 1.00 Reference | ··· |
|  | 41/41,784 (0.10%) | 1.02 (0.75, 1.40) | 0.886 | 1.03 (0.75, 1.42) | 0.835 | 1.02 (0.74, 1.40) | 0.898 | 1.03 (0.75, 1.41) | 0.851 |
| Normal |  |  |  |  |  |  |  |  |  |
|  | 2,718/3,311,635 (0.08%) | 1.00 Reference | ··· | 1.00 Reference | ··· | 1.00 Reference | ··· | 1.00 Reference | ··· |
|  | 169/169,723 (0.10%) | 1.21 (1.04, 1.42) | 0.015 | 1.19 (1.02, 1.39) | 0.028 | 1.21 (1.04, 1.42) | 0.014 | 1.20 (1.02, 1.40) | 0.024 |
| Overweight |  |  |  |  |  |  |  |  |  |
|  | 552/550,696 (0.10%) | 1.00 Reference | ··· | 1.00 Reference | ··· | 1.00 Reference | ··· | 1.00 Reference | ··· |
|  | 33/22,569 (0.15%) | 1.46 (1.03, 2.07) | 0.035 | 1.40 (0.98, 2.00) | 0.061 | 1.46 (1.03, 2.07) | 0.034 | 1.41 (0.99, 1.99) | 0.055 |
| Obesity |  |  |  |  |  |  |  |  |  |
|  | 128/120,306 (0.11%) | 1.00 Reference | ··· | 1.00 Reference | ··· | 1.00 Reference | ··· | 1.00 Reference | ··· |
|  | 8/4,303 (0.19%) | 1.75 (0.86, 3.57) | 0.125 | 1.66 (0.81, 3.41) | 0.167 | 1.75 (0.86, 3.56) | 0.125 | 1.67 (0.82, 3.37) | 0.156 |
| **Perinatal death** |  |  |  |  |  |  |  |  |  |
| Underweight |  |  |  |  |  |  |  |  |  |
|  | 2,108/627,851 (0.34%) | 1.00 Reference | ··· | 1.00 Reference | ··· | 1.00 Reference | ··· | 1.00 Reference | ··· |
|  | 128/41,784 (0.31%) | 0.91 (0.76, 1.09) | 0.313 | 0.89 (0.75, 1.07) | 0.216 | 0.91 (0.76, 1.09) | 0.302 | 0.89 (0.75, 1.07) | 0.220 |
| Normal |  |  |  |  |  |  |  |  |  |
|  | 10,545/3,311,635 (0.32%) | 1.00 Reference | ··· | 1.00 Reference | ··· | 1.00 Reference | ··· | 1.00 Reference | ··· |
|  | 592/169,723 (0.35%) | 1.10 (1.01, 1.19) | 0.031 | 1.05 (0.97, 1.14) | 0.225 | 1.09 (1.00, 1.18) | 0.042 | 1.05 (0.97, 1.14) | 0.235 |
| Overweight |  |  |  |  |  |  |  |  |  |
|  | 2,144/550,696 (0.39%) | 1.00 Reference | ··· | 1.00 Reference | ··· | 1.00 Reference | ··· | 1.00 Reference | ··· |
|  | 107/22,569 (0.47%) | 1.22 (1.00, 1.48) | 0.046 | 1.15 (0.94, 1.40) | 0.164 | 1.21 (1.00, 1.47) | 0.050 | 1.15 (0.95, 1.40) | 0.159 |
| Obesity |  |  |  |  |  |  |  |  |  |
|  | 531/120,306 (0.44%) | 1.00 Reference | ··· | 1.00 Reference | ··· | 1.00 Reference | ··· | 1.00 Reference | ··· |
|  | 21/4,303 (0.49%) | 1.11 (0.71, 1.71) | 0.651 | 1.03 (0.67, 1.60) | 0.884 | 1.10 (0.71, 1.71) | 0.656 | 1.04 (0.67, 1.60) | 0.877 |

Underweight, BMI < 18.5 kg/m^2^; Normal weight, BMI between 18.5 and < 24.0 kg/m^2^; Overweight, BMI between 24.0 and < 28.0 kg/m^2^; Obesity, BMI ≥ 28.0 kg/m^2^.

Model was adjusted for maternal age, ethnicity, educational level, occupation, region, smoking, passive smoking, alcohol consumption, parity, preconception medicine use, folic acid use, hypertension, diabetes, anemia, thyroid disorder, liver disorder, and infection.

Abbreviations: IPTW, inverse probability of treatment weighted; OR, odds ratio; CI, confidence interval; BMI, body mass index.
