# Supplementary material for: Preconception hypoglycemia and adverse pregnancy outcomes in Chinese women aged 20–49 years: A retrospective cohort study in China
Source: PLoS Med. 2025 Jul 29;22(7):e1004667. doi: 10.1371/journal.pmed.1004667 (PMC12306775; doi:10.1371/journal.pmed.1004667)
Supplement: S5 Table — Underweight, BMI < 18.5 kg/m2; Normal weight, BMI between 18.5 and 23.9 kg/m2; Overweight, BMI between 24.0 and 27.9 kg/m2; Obesity, BMI ≥ 28.0 kg/m2. Model was adjusted for maternal age, ethnicity, educational level, occupation, region, smoking, passive smoking, alcohol consumption, parity, preconception medicine use, folic acid use, hypertension, diabetes, anemia, thyroid disorder, liver disorder, and infection. Abbreviations: IPTW, inverse probability treatment weighting; FPG, fasting plasma glucose; RERI, relative excess risk due to interaction; OR, odds ratio; CI, confidence interval; BMI, body mass index. (DOCX) [file pmed.1004667.s008.docx]

**S5 Table. Modification effect of maternal preconception BMI on the association between preconception hypoglycemia and adverse pregnancy outcomes.**

|  | **Unweighted** | | | **IPTW** | | |
| --- | --- | --- | --- | --- | --- | --- |
|  | Hypoglycemia | Normal FPG | RERI | Hypoglycemia | Normal FPG | RERI |
| **Medical abortion** |  |  |  |  |  |  |
| Underweight | 0.91 (0.83, 1.00) | 1.09 (1.07, 1.12) | -0.14 (-0.24, -0.04) | 0.88 (0.81, 0.96) | 1.08 (1.06, 1.11) | -0.08 (-0.27, -0.08) |
| Normal | 0.96 (0.92, 1.00) | 1.00 Reference | - | 0.98 (0.93, 1.02) | 1.00 Reference | - |
| Overweight | 1.04 (0.94, 1.16) | 1.09 (1.07, 1.12) | -0.01 (-0.14, 0.11) | 1.05 (0.95, 1.17) | 1.08 (1.06, 1.10) | -0.01 (-0.13, 0.11) |
| Obesity | 1.21 (0.97, 1.51) | 1.17 (1.12, 1.22) | 0.08 (-0.20, 0.36) | 1.23 (1.04, 1.44) | 1.16 (1.11, 1.22) | 0.09 (-0.12, 0.30) |
| **Miscarriage or early stillbirth** |  |  |  |  |  |  |
| Underweight | 0.98 (0.92, 1.04) | 1.13 (1.11, 1.15) | -0.10 (-0.17, -0.03) | 0.98 (0.92, 1.04) | 1.12 (1.11, 1.14) | -0.10 (-0.17, -0.03) |
| Normal | 0.96 (0.93, 0.99) | 1.00 Reference | - | 0.96 (0.93, 0.99) | 1.00 Reference | - |
| Overweight | 1.03 (0.96, 1.11) | 1.07 (1.05, 1.09) | 0.01 (-0.08, 0.09) | 1.05 (0.96, 1.13) | 1.07 (1.05, 1.09) | 0.02 (-0.06, 0.11) |
| Obesity | 1.23 (1.05, 1.44) | 1.13 (1.09, 1.16) | 0.15 (-0.05, 0.35) | 1.30 (1.16, 1.45) | 1.14 (1.10, 1.18) | 0.20 (0.04, 0.36) |
| **Preterm birth** |  |  |  |  |  |  |
| Underweight | 1.11 (1.07, 1.16) | 1.08 (1.06, 1.09) | -0.08 (-0.13, -0.03) | 1.12 (1.08, 1.16) | 1.10 (1.09, 1.11) | -0.09 (-0.14, -0.04) |
| Normal | 1.12 (1.09, 1.14) | 1.00 Reference | - | 1.10 (1.08, 1.13) | 1.00 Reference | - |
| Overweight | 1.17 (1.11, 1.23) | 1.03 (1.02, 1.04) | 0.02 (-0.04, 0.09) | 1.18 (1.12, 1.23) | 1.03 (1.02, 1.04) | 0.04 (-0.02, 0.11) |
| Obesity | 1.15 (1.02, 1.30) | 1.06 (1.03, 1.08) | -0.02 (-0.16, 0.12) | 1.18 (1.08, 1.29) | 1.05 (1.03, 1.08) | 0.02 (-0.08, 0.13) |
| **Macrosomia** |  |  |  |  |  |  |
| Underweight | 0.67 (0.63, 0.71) | 0.72 (0.71, 0.73) | 0.08 (0.03, 0.12) | 0.66 (0.62, 0.69) | 0.74 (0.74, 0.75) | 0.03 (-0.01, 0.07) |
| Normal | 0.88 (0.85, 0.90) | 1.00 Reference | - | 0.88 (0.86, 0.91) | 1.00 Reference | - |
| Overweight | 1.19 (1.13, 1.27) | 1.36 (1.34, 1.38) | -0.04 (-0.11, 0.04) | 1.18 (1.12, 1.25) | 1.37 (1.36, 1.39) | -0.08 (-0.15, -0.01) |
| Obesity | 1.34 (1.18, 1.52) | 1.55 (1.52, 1.59) | -0.09 (-0.26, 0.09) | 1.38 (1.26, 1.51) | 1.54 (1.50, 1.57) | -0.04 (-0.17, 0.09) |
| **Low birthweight** |  |  |  |  |  |  |
| Underweight | 1.42 (1.29, 1.55) | 1.42 (1.39, 1.46) | -0.11 (-0.25, 0.04) | 1.43 (1.31, 1.56) | 1.42 (1.39, 1.46) | -0.11 (-0.25, 0.03) |
| Normal | 1.10 (1.04, 1.16) | 1.00 Reference | - | 1.11 (1.05, 1.18) | 1.00 Reference | - |
| Overweight | 1.22 (1.07, 1.40) | 1.08 (1.05, 1.12) | 0.04 (-0.13, 0.22) | 1.18 (1.03, 1.35) | 1.09 (1.06, 1.12) | -0.02 (-0.19, 0.15) |
| Obesity | 1.34 (1.00, 1.79) | 1.21 (1.14, 1.29) | 0.03 (-0.37, 0.43) | 1.41 (1.15, 1.73) | 1.23 (1.16, 1.31) | 0.07 (-0.24, 0.37) |
| **Large for gestational age** |  |  |  |  |  |  |
| Underweight | 0.70 (0.67, 0.73) | 0.76 (0.75, 0.77) | 0.07 (0.03, 0.10) | 0.70 (0.68, 0.73) | 0.78 (0.77, 0.79) | 0.04 (0.01, 0.07) |
| Normal | 0.87 (0.86, 0.89) | 1.00 Reference | - | 0.88 (0.86, 0.90) | 1.00 Reference | - |
| Overweight | 1.11 (1.06, 1.16) | 1.31 (1.30, 1.32) | -0.07 (-0.12, -0.02) | 1.10 (1.06, 1.15) | 1.33 (1.32, 1.34) | -0.11 (-0.16, -0.06) |
| Obesity | 1.21 (1.10, 1.34) | 1.44 (1.42, 1.47) | -0.10 (-0.23, 0.02) | 1.23 (1.15, 1.32) | 1.43 (1.41, 1.46) | -0.08 (-0.18, 0.01) |
| **Small for gestational age** |  |  |  |  |  |  |
| Underweight | 1.45 (1.40, 1.50) | 1.40 (1.38, 1.41) | -0.01 (-0.06, 0.04) | 1.49 (1.44, 1.53) | 1.38 (1.37, 1.40) | 0.02 (-0.04, 0.07) |
| Normal | 1.06 (1.04, 1.08) | 1.00 Reference | - | 1.09 (1.06, 1.11) | 1.00 Reference | - |
| Overweight | 0.94 (0.89, 1.00) | 0.85 (0.84, 0.86) | 0.03 (-0.02, 0.09) | 0.95 (0.90, 1.00) | 0.86 (0.85, 0.87) | 0.01 (-0.05, 0.06) |
| Obesity | 0.97 (0.85, 1.10) | 0.83 (0.81, 0.85) | 0.07 (-0.05, 0.20) | 0.97 (0.88, 1.06) | 0.86 (0.83, 0.88) | 0.02 (-0.07, 0.12) |
| **Birth defects** |  |  |  |  |  |  |
| Underweight | 1.20 (0.88, 1.63) | 1.17 (1.07, 1.28) | -0.17 (-0.59, 0.25) | 1.09 (0.80, 1.49) | 1.17 (1.08, 1.26) | -0.32 (-0.72, 0.08) |
| Normal | 1.20 (1.02, 1.40) | 1.00 Reference | - | 1.24 (1.05, 1.46) | 1.00 Reference | - |
| Overweight | 1.64 (1.16, 2.31) | 1.14 (1.04, 1.26) | 0.30 (-0.30, 0.89) | 1.47 (1.03, 2.10) | 1.15 (1.06, 1.26) | 0.08 (-0.48, 0.64) |
| Obesity | 2.03 (1.01, 4.08) | 1.19 (1.00, 1.43) | 0.64 (-0.79, 2.08) | 2.04 (1.22, 3.42) | 1.25 (1.05, 1.50) | 0.55 (-0.54, 1.64) |
| **Perinatal death** |  |  |  |  |  |  |
| Underweight | 0.97 (0.81, 1.16) | 1.09 (1.04, 1.15) | -0.17 (-0.37, 0.02) | 0.94 (0.80, 1.12) | 1.09 (1.04, 1.13) | -0.19 (-0.38, -0.01) |
| Normal | 1.05 (0.97, 1.15) | 1.00 Reference | - | 1.05 (0.96, 1.15) | 1.00 Reference | - |
| Overweight | 1.31 (1.08, 1.59) | 1.14 (1.08, 1.19) | 0.11 (-0.15, 0.38) | 1.34 (1.12, 1.61) | 1.13 (1.09, 1.19) | 0.16 (-0.11, 0.42) |
| Obesity | 1.30 (0.84, 1.99) | 1.24 (1.14, 1.36) | 0.00 (-0.58, 0.57) | 1.35 (0.99, 1.86) | 1.27 (1.16, 1.40) | 0.03 (-0.43, 0.48) |

Underweight, BMI < 18.5 kg/m^2^; Normal weight, BMI between 18.5 and < 24.0 kg/m^2^; Overweight, BMI between 24.0 and < 28.0 kg/m^2^; Obesity, BMI ≥ 28.0 kg/m^2^.

Model was adjusted for maternal age, ethnicity, educational level, occupation, region, smoking, passive smoking, alcohol consumption, parity, preconception medicine use, folic acid use, hypertension, diabetes, anemia, thyroid disorder, liver disorder, and infection.

Abbreviations: IPTW, inverse probability of treatment weighted; FPG, fasting plasma glucose; RERI, relative excess risk due to interaction; OR, odds ratio; CI, confidence interval; BMI, body mass index.
